# Dissecting the low catalytic capability of flavin-dependent halogenases

**DOI:** 10.1074/jbc.RA120.016004

**Published:** 2020-11-23

**Authors:** Aisaraphon Phintha, Kridsadakorn Prakinee, Aritsara Jaruwat, Narin Lawan, Surawit Visitsatthawong, Chadaporn Kantiwiriyawanitch, Warangkhana Songsungthong, Duangthip Trisrivirat, Pirom Chenprakhon, Adrian Mulholland, Karl-Heinz van Pée, Penchit Chitnumsub, Pimchai Chaiyen

**Affiliations:** 1 Department of Biochemistry and Center for Excellence in Protein and Enzyme Technology, Faculty of Science, Mahidol University, Bangkok, Thailand; 2 School of Biomolecular Science and Engineering, Vidyasirimedhi Institute of Science and Technology (VISTEC), Rayong, Thailand; 3 Biomolecular Analysis and Application Research Team, National Center for Genetic Engineering and Biotechnology, Klong Luang, Pathumthani, Thailand; 4 Department of Chemistry, Faculty of Science, Chiang Mai University, Chiang Mai, Thailand; 5 Institute for Innovative Learning, Mahidol University, Nakhon Pathom, Thailand; 6 Centre for Computational Chemistry, School of Chemistry, University of Bristol, Bristol, UK; 7 General Biochemistry, Faculty of Chemistry and Food Chemistry, Technical University of Dresden, Dresden, Germany

**Keywords:** flavin monooxygenase, halogenase, X-ray structures, kinetics, stopped-flow, QM/MM calculations, C_1_, the flavin reductase component of *p*-hydroxyphenylacetate 3-hydroxylase from *Acinetobacter baumannii*, DAD, diode array detector, DMSO-_*d6*_, Methyl sulfoxide-_*d6*_, FAD, oxidized flavin adenine dinucleotide, FADH^–^, reduced flavin adenine dinucleotide, HCOONa, sodium formate, HOBr, hypobromous acid, HOCl, hypochlorous acid, HOI, hypoiodous acid, HOX, hypohalous acid, HPLC, high-performance liquid chromatography, MS, mass spectrometry, Na_2_S_2_O_4_, sodium dithionite, NAD^+^, nicotinamide adenine dinucleotide, PrnA, tryptophan 7-halogenase from *Pseudomonas fluorescens*, psFDH, formate dehydrogenase from *Pseudomonas sp.*, PyrH, tryptophan 5-halogenase from *Streptomyces rugosporus*, QM/MM, quantum mechanics/molecular mechanics, Thal, tryptophan 6-halogenase from *Streptomyces albogriseolus*

## Abstract

Although flavin-dependent halogenases (FDHs) are attractive biocatalysts, their practical applications are limited because of their low catalytic efficiency. Here, we investigated the reaction mechanisms and structures of tryptophan 6-halogenase (Thal) from *Streptomyces albogriseolus* using stopped-flow, rapid-quench flow, quantum/mechanics molecular mechanics calculations, crystallography, and detection of intermediate (hypohalous acid [HOX]) liberation. We found that the key flavin intermediate, C4a-hydroperoxyflavin (C4aOOH-FAD), formed by Thal and other FDHs (tryptophan 7-halogenase [PrnA] and tryptophan 5-halogenase [PyrH]), can react with I^−^, Br^−^, and Cl^−^ but not F^−^ to form C4a-hydroxyflavin and HOX. Our experiments revealed that I^−^ reacts with C4aOOH-FAD the fastest with the lowest energy barrier and have shown for the first time that a significant amount of the HOX formed leaks out as free HOX. This leakage is probably a major cause of low product coupling ratios in all FDHs. Site-saturation mutagenesis of Lys79 showed that changing Lys79 to any other amino acid resulted in an inactive enzyme. However, the levels of liberated HOX of these variants are all similar, implying that Lys79 probably does not form a chloramine or bromamine intermediate as previously proposed. Computational calculations revealed that Lys79 has an abnormally lower p*K*a compared with other Lys residues, implying that the catalytic Lys may act as a proton donor in catalysis. Analysis of new X-ray structures of Thal also explains why premixing of FDHs with reduced flavin adenine dinucleotide generally results in abolishment of C4aOOH-FAD formation. These findings reveal the hidden factors restricting FDHs capability which should be useful for future development of FDHs applications.

Halogenation is extremely important for the agricultural, pharmaceutical, and chemical industries (1). It is especially of importance for drug synthesis because one-third of currently produced drugs, particularly new drugs currently in clinical trials are halogenated compounds (2, 3, 4, 5, 6, 7, 8, 9). However, by traditional chemical synthesis, halogenations are generally not environmentally friendly reactions because most of these reactions need to be carried out under harsh conditions and often involve the use of toxic reagents, catalysts, and solvents. Chemical halogenation reactions also lack regioselectivity, resulting in many by-products (10, 11, 12, 13). The use of enzymes, *i.e.*, halogenases, to catalyze halogenation is an attractive green chemistry method because the reactions can be done under mild conditions. Halogenases discovered to date can be classified into four groups: haloperoxidases, fluorinases, α-ketoglutarate-dependent halogenases, and flavin-dependent halogenases (FDHs) (14).

FDHs belong to the class of two-component flavin-dependent monooxygenases which require a reductase component to generate reduced FAD (FADH^−^) for a halogenase (monooxygenase) component to catalyze halogenation (14, 15, 16, 17, 18, 19). Several X-ray structures of FDHs have been solved (18) which showed that all FDHs have two separated binding sites. While the FAD binding site is well conserved among different FDHs, a significant difference lies in the substrate binding site which allows FDHs to utilize a broad range of substrates and catalyze halogenation at various positions (14). Therefore, most of the previous studies have been focused on enzyme engineering to alter substrate scope and regio-specificity (14, 20, 21, 22, 23, 24, 25, 26), whereas less attention has been paid to mechanistic investigation.

To date, mechanistic investigation of FDHs has only been carried out with the reactions of tryptophan 7-halogenases (RebH (15, 27, 28) and PrnA (29)) with only one investigation of transient kinetics of RebH reported (27, 28). The results indicate that RebH can form a C4a-hydroperoxyflavin intermediate (C4aOOH-FAD) to react with Br^−^ and Cl^−^ (the reactions with I^−^ and F^−^ were not explored) to generate a reactive halogenation species (28), hypohalous acid (HOX) (hypobromous acid [HOBr] and hypochlorous acid [HOCl]). HOX is thought to be transferred to a second active site to react with tryptophan (27, 28, 29). Because site-directed mutagenesis of the conserved Lys to Ala (15, 30, 31) and to Arg (31) resulted in inactive tryptophan halogenases, two models to explain the mechanism of halogenation have been proposed. In the first model, HOX is thought to react with Lys to form the chloramine intermediate which acts as a halogenating species (27), whereas in the second model, HOX is thought to be a direct halogenation reagent with the conserved Lys facilitating and controlling the binding of HOX in the proper geometry necessary for halogenation (15, 32). Interestingly, none of the studies since then have investigated the function of the conserved Lys. None of the previous studies has also investigated the mechanism of HOX formation and determined the factors controlling HOX production. Most FDHs are inefficient because their turnovers are quite slow (*k*_cat_ = 0.5–3 min^−1^), and they can be easily inactivated (26). Despite gram scale synthesis of halogenated tryptophan product was reported for the reaction of RebH, full conversion was obtained only after 8 days (33), which is too slow to be accepted for a real industrial process. Therefore, kinetic and structural investigation to understand mechanistic features controlling their halogenation are necessary for future improvement of FDHs for industrial applications.

Tryptophan 6-halogenase or Thal from *Streptomyces albogriseolus* (34, 35) represents an interesting FDH system for investigating the mechanisms of HOX formation and factors controlling halogenation. As Thal catalyzes tryptophan halogenation at the 6-position, not 7-position as in RebH or PrnA, the results would also contribute to the understanding of a mechanism related to halogenation of tryptophan at the 6-position. Recently, crystal structures of Thal were solved and the results showed that FAD and tryptophan substrates could not be co-crystallized (34). This feature is different from other tryptophan halogenases such as RebH (36), tryptophan 5-halogenase (PyrH) (37) and PrnA (15) which could be co-crystallized with both FAD and tryptophan. This suggests that protein dynamics between the flavin and substrate binding sites of Thal are strongly linked. Therefore, we set out to investigate the mechanism of HOX formation and its role in regulating the Thal reaction and used Thal as a model for exploring the factors contributing to the catalytic inefficiency of FDHs in general.

In this report, the reaction of Thal was investigated with regard to four main mechanistic issues to unravel why the reactions of FDHs are not very efficient. (A) The mechanism of HOX formation was investigated using transient kinetics (stopped-flow absorbance and fluorescence and rapid-quench flow techniques) in combination with quantum/mechanics molecular mechanics (QM/MM) computational calculations. (B) The cause of the low product coupling ratio was investigated by measuring the leakage of the HOX intermediate using an organic substrate (D-luciferin) as a halogenating target to detect free HOX. (C) The functional role of the conserved Lys was investigated using site-saturation mutagenesis studies along with detection of free HOX formation and theoretical p*K*a calculations. Unlike the mechanism previously proposed, our results do not support formation of chloramine or bromamine intermediates but rather suggest that the conserved Lys likely protonates the HOX in the halogenation reaction. (D) Results from transient kinetics, newly crystallized structures of Thal:FADH^−^ and Thal:FAD:AMP and molecular dynamics (MD) simulations could be used to explain the cause for formation of the dead-end inactive Thal:FADH^-^^∗^ complex.

## Results

### Halogenation activity of Thal

To explore whether Thal represents a halogenase which can catalyze a reasonable halogenation reaction, multiple turnover reactions of Thal were carried out and compared with the reactions of PrnA and PyrH. The products from the Thal reaction were characterized by NMR spectroscopy (Fig. S2*C*). The results indicated that among the three halogenases investigated, Thal was the fastest halogenase (Fig. S2*A*). These data also clearly imply that most of the halogenases are not very efficient enzymes which agrees with previous data reporting that FDHs suffer from having low *k*_cat_ values (26). Therefore, understanding the cause of catalytic inefficiency in Thal as a representative of FDHs would contribute to identification of the bottleneck in the reaction of FDHs in general.

**Figure 1:**
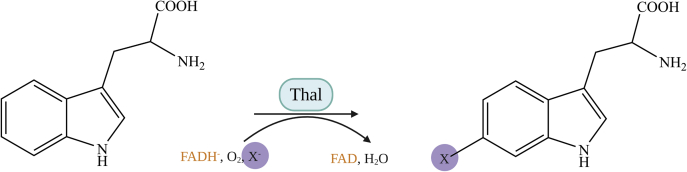
**Reaction of Thal with tryptophan.** Thal catalyzes halogenation of tryptophan to yield halogenated tryptophan as the product. FAD, oxidized flavin adenine dinucleotide; FADH^−^, reduced flavin adenine dinucleotide.

The percentage of product halogenation in the Thal reaction was measured from the consumption of the substrate (tryptophan) in single turnover reactions carried out in a rapid-quench flow instrument (Experimental Procedures). The reaction of Thal was carried out using FADH^−^ as a limiting reagent (Fig. 1). After the first turnover was completed (data collected at 400 s), we found that only about 30% of the tryptophan was halogenated (data not shown). The results indicate that the coupling ratio (product produced per substrate consumed) is quite low, which is likely one of the factors explaining the catalytic inefficiency in Thal and in FDHs in general. Therefore, we further explored the fates of each reaction intermediate after C4aOOH-FAD is formed.

### Determination of the hydrogen peroxide elimination ratio from the uncoupling (nonproductive) path

**Figure 2:**
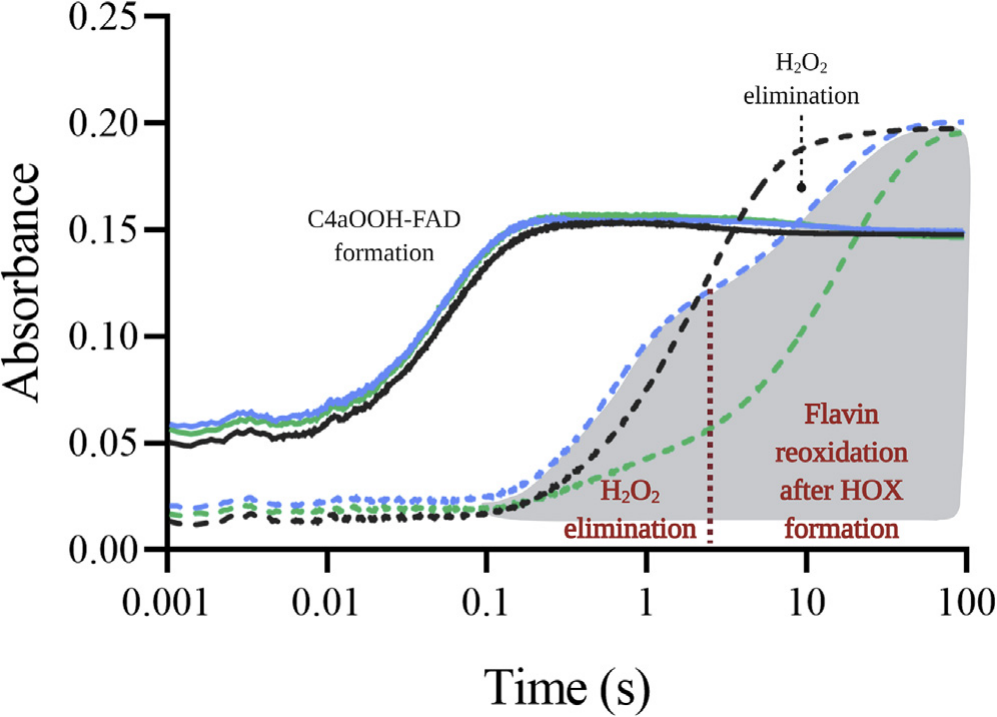
**Kinetic traces of the Thal reaction in the presence of halide ions.** An air-saturated solution of Thal premixed with NaCl (*blue lines*) or NaBr (*green lines*) was mixed with an anaerobic solution of FADH^−^. *Black lines* are kinetic traces without addition of halide ions. Flavin intermediates were monitored by absorption changes at 380 nm (*solid lines*) and 450 nm (*dashed lines*). C4aOOH-FAD, C4a-hydroperoxyflavin; FADH^−^, reduced flavin adenine dinucleotide.

A stopped-flow experiment was carried out under conditions in which C4aOOH-FAD was fully formed (details described in Figs. S5 and S6). In the absence of halide, mixing of an anaerobic solution of FADH^−^ with an aerobic solution of Thal resulted in reactions with kinetic traces showing two phases (Fig. 2). The first phase (0.02–0.15 s) showed an increase in absorbance at 380 nm with almost no change at 450 nm. The second phase (0.15–100 s) showed a decrease in absorbance at 380 nm which was concomitant with an increase in absorbance at 450 nm. Kinetic traces of the first phase and second phase represent C4aOOH-FAD formation (*k*_obs_ of 16.52 s^−1^) and decay (*k*_obs_ of 0.41 s^−1^) to form hydrogen peroxide (H_2_O_2_) and FAD, respectively (Fig. 2).

The kinetics of C4aOOH-FAD formation in the presence of 10 mM Br^−^ or Cl^−^ (the condition in which HOBr or HOCl was formed maximally, as explained in detail in the next section) was the same as that in the absence of Br^−^ or Cl^−^ (Fig. 2), but the kinetics of the reaction after C4aOOH-FAD formation was different. The reaction in the presence of halide ions showed three phases. The first phase (0.02–0.15 s) showed an increase in absorbance at 380 nm. The second (0.15–2.5 s) and third phases (2.5–100 s) showed a decrease in absorbance at 380 nm and an increase in absorbance at 450 nm. The kinetics of the second phase of both reactions (*k*_obs_ of 1.6 s^−1^) were similar to that in the absence of halide, indicating that this phase was the uncoupling path to elimination of H_2_O_2_ without forming HOBr or HOCl. As the amplitude of this phase (absorbance 450 nm) in the presence of Br^−^ and Cl^−^ was about 20% and 55%, respectively, the results indicate that the uncoupling path is more prominent in the presence of Cl^−^ compared with Br^−^ and that C4aOOH-FAD reacts more efficiently with Br^−^ than Cl^−^.

The kinetics of the third phase was clearly different from the reaction in the absence of the halide ions. This phase was later identified as the step of C4a-hydroxyflavin (C4aOH-FAD) decay after HOX formation (see the following results in the next section). These data indicate that even under saturating amounts of halide, the reaction of Thal still proceeds through an uncoupling path (no formation of HOX) with a frequency of about 20% and 55% in the case of Br^−^ and Cl^−^, respectively. The data also indicate that 80% of the C4aOOH-FAD intermediate can react with Br^−^ to form HOBr. However, our results in the first section (halogenation activity of Thal) indicate that only 30% of the tryptophans were brominated, implying that not all of the HOBr formed reacts with tryptophan. We therefore investigated the mechanisms of HOX formation by FDHs and examined their control of HOX reactivity.

### Mechanism of HOX formation

#### Kinetics of hypohalous acid (HOCl, HOBr, and hypoiodous acid) formation catalyzed by Thal, PrnA, and PyrH

Because the reaction of halide ions with C4aOOH-FAD is crucial for the catalysis of FDHs and this reaction is not well understood, we investigated the reactivity of halide ions with Thal and other tryptophan halogenases (PrnA and PyrH) by monitoring the kinetics of its formation.

**Figure 3:**
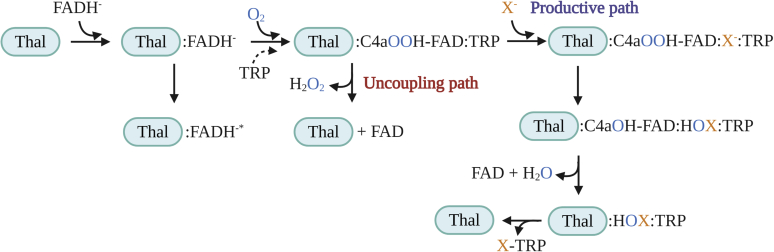
**Kinetic mechanisms of the Thal reaction with tryptophan.** FAD refers to oxidized FAD. FADH^−^ refers to reduced FAD. Thal:FADH^-^^∗^ refers to inactive complex which cannot form C4aOOH-FAD. TRP refers to tryptophan. C4aOOH-FAD refers to C4a-hydroperoxyflavin. C4aOH-FAD refers to C4a-hydroxyflavin. X^−^ represents the halide ions. HOX represents hypohalous acid. In this model, TRP can bind to Thal together with a flavin.

**Figure 4:**
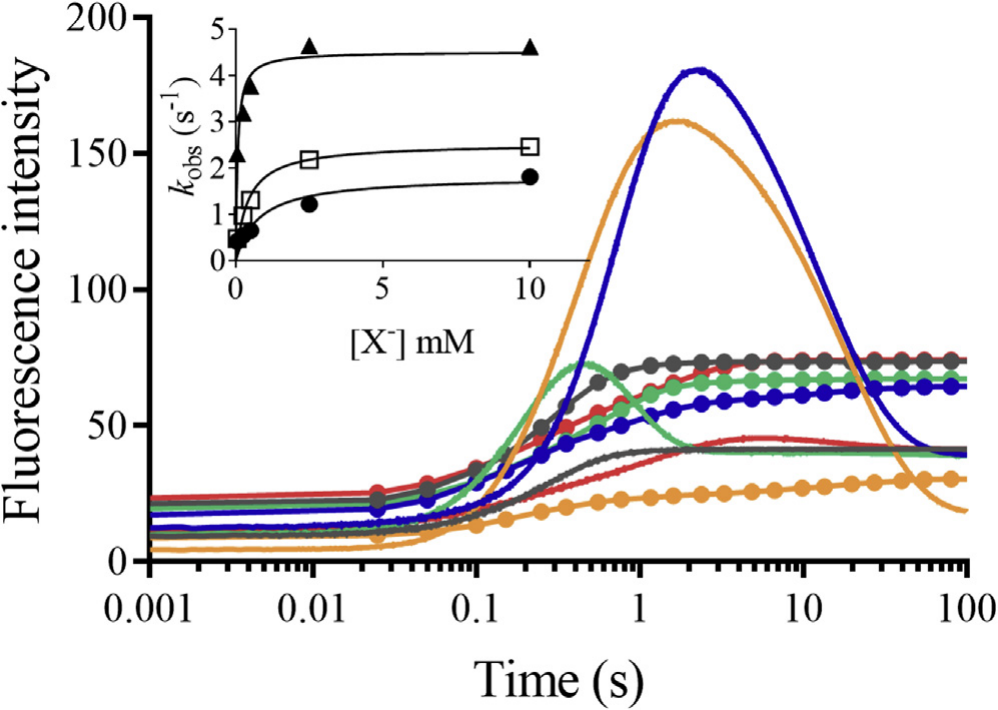
**Kinetic traces of the Thal reaction with various halide ions.** Formation of C4aOH-FAD was monitored at Em > 495 nm using excitation wavelengths of 380 nm (*solid lines*) and 450 nm (*solid lines with circles*) in the presence of various halide ions (Cl^−^ (*blue*), Br^−^ (*orange*), I^−^ (*green*), and F^−^ (*red*)). *Gray* lines are reactions without addition of halide ion. Note that the difference in fluorescence intensities was because of the difference of photomultiplier tube voltage used for the various halide reactions. Inset shows a plot of the observed rate constants of C4aOH-FAD formation resulting from the reaction of C4aOOH-FAD with halide ions (Cl^−^ (*circle*), Br^−^ (*square*), and I^−^ (*triangular*)) at various concentrations. C4aOOH-FAD, C4a-hydroperoxyflavin; C4aOH-FAD, C4a-hydroxyflavin; Em, emission wavelength.

To monitor the kinetics of HOX formation, we monitored the transitions from C4aOOH-FAD to C4aOH-FAD and HOX (Fig. 3). C4aOH-FAD is a common intermediate in the reactions of flavin-dependent monooxygenases (28, 38, 39) and can be distinguished from C4aOOH-FAD by its strong fluorescence in several enzymes such as the flavin-dependent dehalogenase and 2-methyl-3-hydroxypyridine 5-carboxylic acid monooxygenase (38, 40, 41). Therefore, we carried out similar experiments as in the previous section but instead we measured the appearance of highly fluorescent species using excitation wavelengths (Ex) of 380 nm and 450 nm and monitoring emission wavelength at > 495 nm. Results in Figure 4 show a large increase in fluorescence during 0.15 to 2.5 s (corresponding to an observed rate constant of 1.5 s^−1^) in the presence of Cl^−^ when using Ex at 380 nm (blue solid line). This fluorescent species decayed during 2.5 to 100 s (corresponding to an observed rate constant of 0.073 s^−1^). The data clearly indicate that the fluorescence decay occurred simultaneously with the third phase of Figure 2 in which the absorbance at 380 nm decreased and the absorbance at 450 nm increased. The data in Figures 2 and 4 indicate that in the presence of Cl^−^ after C4aOOH-FAD was formed, and the next step was formation of a highly fluorescent C4aOH-FAD species which eventually eliminates H_2_O to form oxidized FAD (Fig. 3). The conversion of C4aOOH-FAD to C4aOH-FAD was then used for measuring rate constants of HOX formation in the reactions of various FDHs.

To explore whether other halide ions (F^−^, I^−^, and Br^−^) can also react with C4aOOH-FAD in the Thal reaction, reactions of Thal with various halide ions were carried out. Results in Figure 4 indicate that C4aOOH-FAD in Thal can react with I^−^, Br^−^, and Cl^−^ to form a highly fluorescent C4aOH-FAD intermediate. The exception was found for F^−^ in which a highly fluorescent flavin could not be detected. These results clearly indicate that Thal can catalyze the reaction of C4aOOH-FAD with I^−^, Br^−^, and Cl^−^ to form C4aOH-FAD and hypoiodous acid (HOI), HOBr, and HOCl, respectively, and it is the first evidence to show formation of HOI in the reaction of an FDH. Previously, the reaction of RebH was only investigated in regard to its ability to form HOBr and HOCl.

To explore whether the reactivity of Thal to form HOI is only specific to this enzyme, we also examined the reactivity of other halogenases such as PrnA and PyrH with I^−^. Similar experiments to those in Figure 4 were carried out. The results (Fig. S7) clearly showed that PrnA and PyrH could also react with I^−^ to form HOI and C4aOH-FAD. However, product analysis indicates that Thal could not catalyze iodination of tryptophan (Fig. S8), indicating that the factor limiting iodination of tryptophan is not on the flavin reaction site (described in more detail using crystal structures of Thal in the last section).

#### Kinetic constants of the reaction of C4aOOH-FAD with halide to form C4aOH-FAD and HOX

To measure rate constants associated with kinetics of HOX formation, the reactions of air-saturated solutions of Thal containing various concentrations of NaI, KBr, or NaCl were mixed with a solution of FADH^−^. An increase in fluorescence because of formation of C4aOH-FAD as described in the determination of H_2_O_2_ elimination section was used for analysis of kinetics of HOX formation. Plots of *k*_obs_ *versus* halide ion concentrations (inset of Fig. 4) showed hyperbolic relationships which indicate that the reaction involves at least two steps. The analysis suggests that the first step is the binding of a halide to C4aOOH-FAD to form C4aOOH-FAD:X^−^ which is then converted to yield C4aOH-FAD and HOX in the second step (Fig. 3). Kinetics of the reaction of halide with C4aOOH-FAD was analyzed according to a model describing a two-step reaction (Fig. 4) (42). Analysis of the data in Figure 4 is summarized in Table 1. The data indicate that I^−^ showed the fastest rate in the reaction to form C4aOH-FAD followed by Br^−^ and Cl^−^, respectively. These results could be explained using QM/MM calculations to calculate the energy associated with the reactions (see QM/MM results later).

**Table 1:** Reactivity of halide ions with C4aOOH-FAD to form C4aOH-FAD

Halide ion	K_d_ (mM)	*k* (s^−1^)
I^−^	0.064 ± 0.021	4.51 ± 0.24
Br^−^	0.40 ± 0.073	2.54 ± 0.12
Cl^−^	0.78 ± 0.39	1.83 ± 0.26

C4aOOH-FAD, C4a-hydroperoxyflavin; C4aOH-FAD, C4a-hydroxyflavin.

#### QM/MM calculations to explain the reactivity of C4aOOH-FAD with different halide ions

To investigate the reaction mechanism of HOX formation at a molecular and electronic level, we performed *ab initio* QM/MM calculations using SCS-MP2/aug-cc-pvdz, I=auc-cc-pwcvdz-pp/CHARMM27 (Experimental Procedures) to compare with rate constants obtained from the experimental results. Our experiments in the previous section showed that I^−^ reacts with C4aOOH-FAD with the highest rate constant, followed by Br^−^ and Cl^−^, respectively (Table 1, Fig. 4). This level of QM/MM theory has been shown to give accurate barriers for other enzyme-catalyzed reactions (43, 44, 45, 46). The proposed reaction mechanism of HOCl formation in FDHs is shown in Fig. S9. The overall reaction is composed of two steps: (i) C4aOOH-FAD-Cl formation, corresponding to Cl-OO bond forming (R_Cl-O2_) and (ii) HOCl release, corresponding to O1-O2 bond breakage (R_O1-O2_). Potential energy surfaces of SCS-MP2/aug-cc-pvdz/CHARMM27 calculations for this reaction are shown in Fig. S13. The Cl^−^ attacks the -OO- atom of C4aOOH-FAD to form C4aOOH-FAD-Cl along the R_Cl-O2_. Along the R_O1-O2_ which is the release of HOCl, the O1-O2 bond is broken. The potential energy increase was calculated between the reactant (0.0 kcal/mol) and the transition state levels, giving an activation energy of 12.6 kcal/mol. The products were calculated to be significantly lower in energy than the reactant, −28.5 kcal/mol. Potential energy surfaces for HOBr and HOI formation were also calculated, and the results are shown in Figs. S14 and S15, respectively. The calculated reaction energetics (relative potential energies of reactants, transition state, and products) are summarized in Table 2. Structural and energetic details of the key species in the reaction are given in Supporting Information. The results clearly indicate that the energy barrier to HOX formation for the different halides can be ranked as HOCl > HOBr > HOI, consistent with the rate constants of HOX formation as measured by stopped-flow experiments (Fig. 4) as HOI > HOBr > HOCl.

**Table 2:** Potential energies of various states for HOX (X = I, Br, or Cl) formation catalyzed by Thal

Formation of	Potential energy (kcal/mol)		
	Reactant	TS	Product
HOI	0.0	1.3	−51.8
HOBr	0.0	5.7	−39.8
HOCl	0.0	12.6	−28.5

HOBr, hypobromous acid; HOCl, hypochlorous acid; HOI, hypoiodous acid; HOX, hypohalous acid.

### Investigation of hypohalous acid leakage

**Figure 5:**
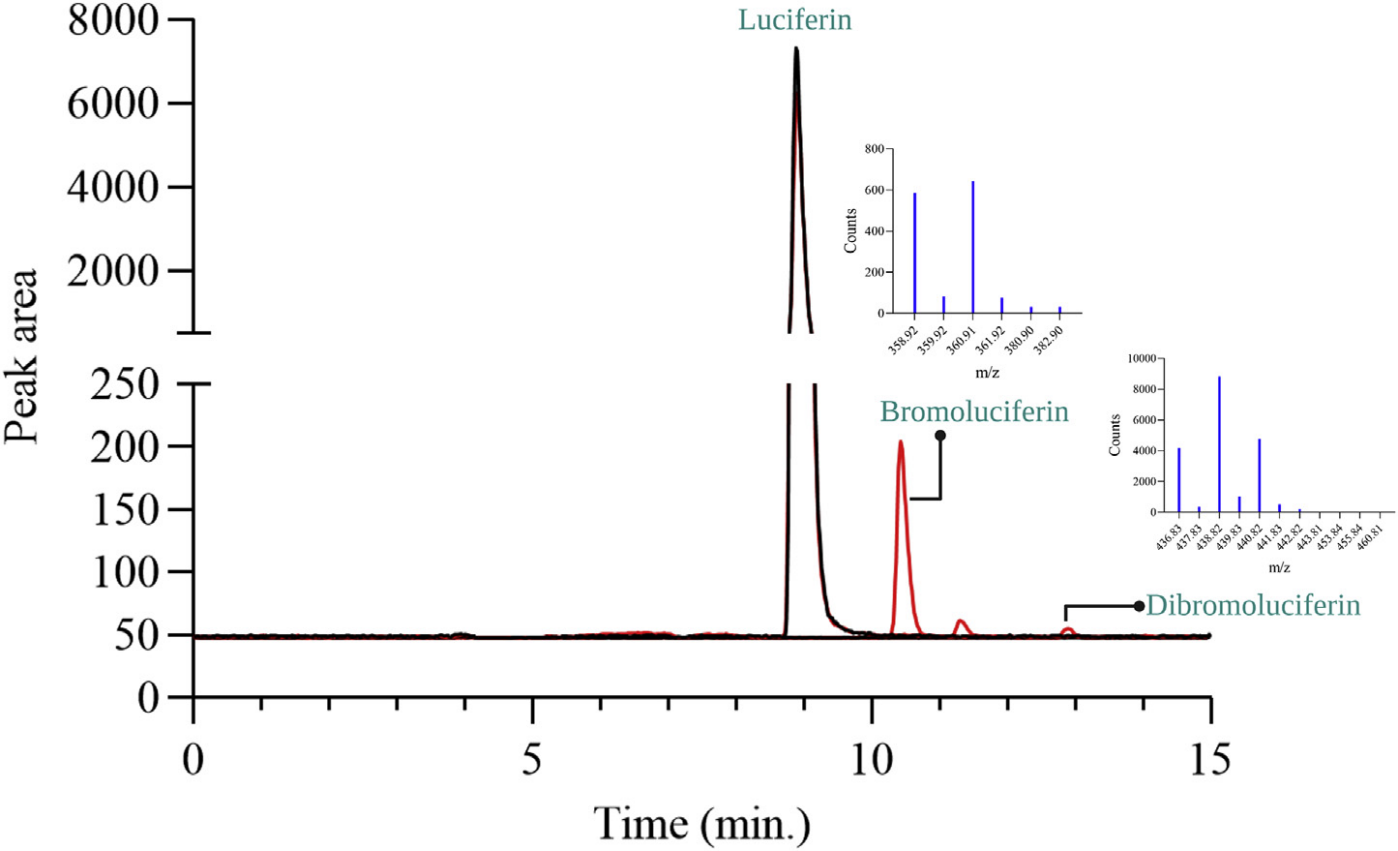
**Detection of HOBr leakage from Thal (wild-type).** HPLC-MS/MS chromatograms of the reaction without Thal (*black*) and the reaction in the presence of Thal (*red*). Multiple turnover reactions were performed to generate HOBr. A reaction solution after removal of enzymes was incubated with D-luciferin. Reactions were analyzed using HPLC-MS/MS. Mass spectra of bromo-D-luciferin and dibromo-D-luciferin were analyzed using HPLC-QTOF (inset). HOBr, hypobromous acid; QTOF, Quadrupole Time-of-Flight.

As the percentage of product uncoupling (70%) (halogenation activity section) is greater than H_2_O_2_ elimination (20%) (H_2_O_2_ elimination section), we suspected that the generated HOX might be leaked as a free halogenating reagent without being transported through a path between the two active sites to halogenate tryptophan. Previously, investigation of HOX leakage was only carried out in the PrnA system using monochlorodimedone for detection. In this system, free HOX could not be detected (15). In this current work, to investigate whether HOX generated by Thal is efficiently trapped inside the active site or leaks out freely, we used a different method of detection. D-luciferin, a compound susceptible to bromination by HOBr, was used instead of monochlorodimedone for detection of HOBr leakage in multiple turnover reactions of Thal. Products were analyzed using high-resolution mass spectrometry and Quadrupole Time-of-Flight (QTOF) (Experimental Procedures). Results in Figure 5 clearly showed that a significant amount of brominated D-luciferin was detected, indicating that in the wild-type enzyme reaction, free HOBr leaks out from the active site of Thal. The leakage of HOX is probably one of the factors which make Thal and other FDHs ineffective enzymes in general.

### Catalytic function of the conserved Lys

#### Investigation of the functional role of Lys79 using site-saturation mutagenesis

As the conserved catalytic Lys (Lys79^Thal^) is present in all FDHs reported and only two variants of the catalytic Lys (substitution of Lys to Ala (15, 27) or Arg (31)) were reported in the past, we subsequently mutated Lys79^Thal^ to each of the other 19 amino acids using a site-saturation mutagenesis approach (Experimental Procedures) to investigate whether halogenation can also be achieved by other types of amino acids in this position. As the results in the previous section indicate that HOX can leak out from the wild-type enzyme, we also wanted to explore whether changing Lys79 to other residues could change the level of free HOX leakage. If Lys79 can form the chloramine or bromamine as previously proposed, exchanging this residue with other amino acids would generate significantly higher amounts of free HOX.

Plasmid libraries for site-saturation mutagenesis were constructed and evaluated to confirm that they generated all 20 amino acids in the Lys79 position, and a total 168 clones of Lys79 variants were tested for their ability to halogenate tryptophan (Experimental Procedures). The activity of the variants was analyzed by detecting the bromotryptophan product using HPLC-MS/MS. The protein production level of each variant was also measured using a GFP split system (47, 48). The results showed that all of the variants were completely inactive for tryptophan bromination (data not shown). These data confirm that Lys79 is crucial for the Thal reaction and cannot be replaced by another amino acid. One of the inactive variants, Lys79Thr, which expressed well was selected for further mechanistic investigation to understand the catalytic role of Lys79 (Fig. S18).

#### Investigation of C4aOOH-FAD formation by Lys79Thr

**Figure 6:**
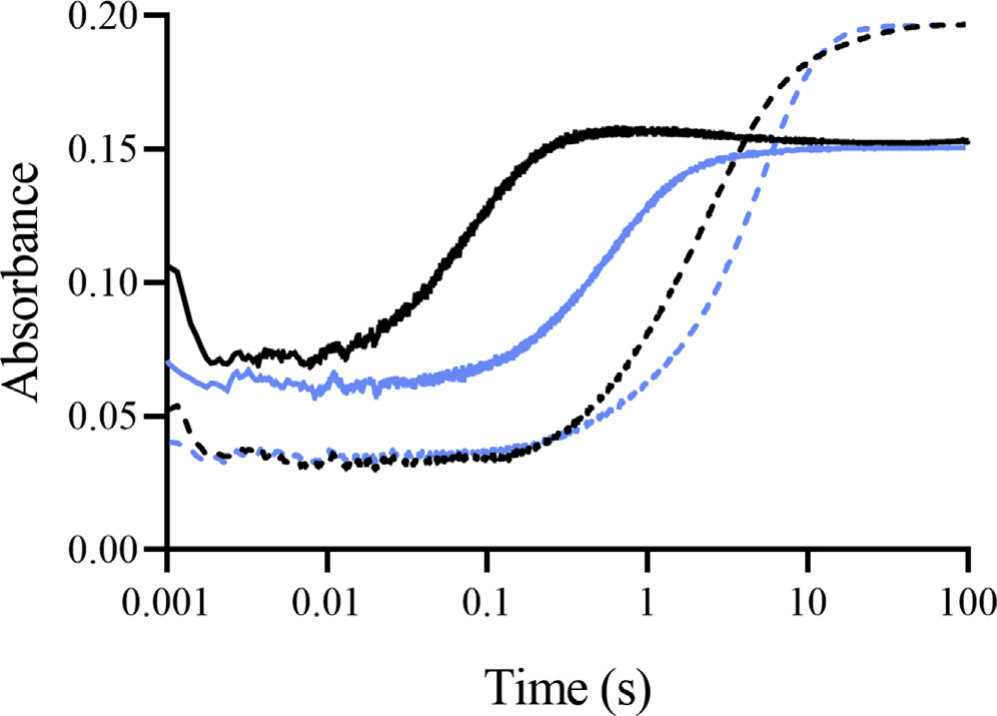
**Kinetics of Lys79Thr reacting with oxygen.** An air-saturated solution of Lys79Thr (*blue lines*) or wild-type (*black lines*) (30 μM) was mixed with a solution of FADH^−^ (15 μM) using a single mixing stopped-flow spectrometer. Kinetic traces were monitored at 380 nm (*solid lines*) and 450 nm (*dashed lines*). FADH^−^, reduced flavin adenine dinucleotide.

To investigate whether the absence of halogenation activity of Lys79Thr is due to the impairment of formation of flavin intermediates, the ability of Lys79Thr to form C4aOOH-FAD was analyzed using stopped-flow spectrometry similar to the experiments carried out for the wild-type enzyme (Fig. 2). Stopped-flow data in Figure 6 showed that an increase in absorbance at 380 nm (indicative of C4aOOH-FAD formation) occurred similarly during the first phase in both the Lys79Thr (*blue lines*, 0.02–0.14 s) and wild-type (*black lines*, 0.02–0.3 s) enzymes. These data indicate that Lys79Thr could still form C4aOOH-FAD, suggesting that inactivation of Lys79Thr is not because of a loss of the ability to form flavin intermediates but must be related to the tryptophan halogenation step. These data confirm that Lys79 has an important role in halogenation.

#### Measurement of HOX leakage by Lys79Thr

**Figure 7:**
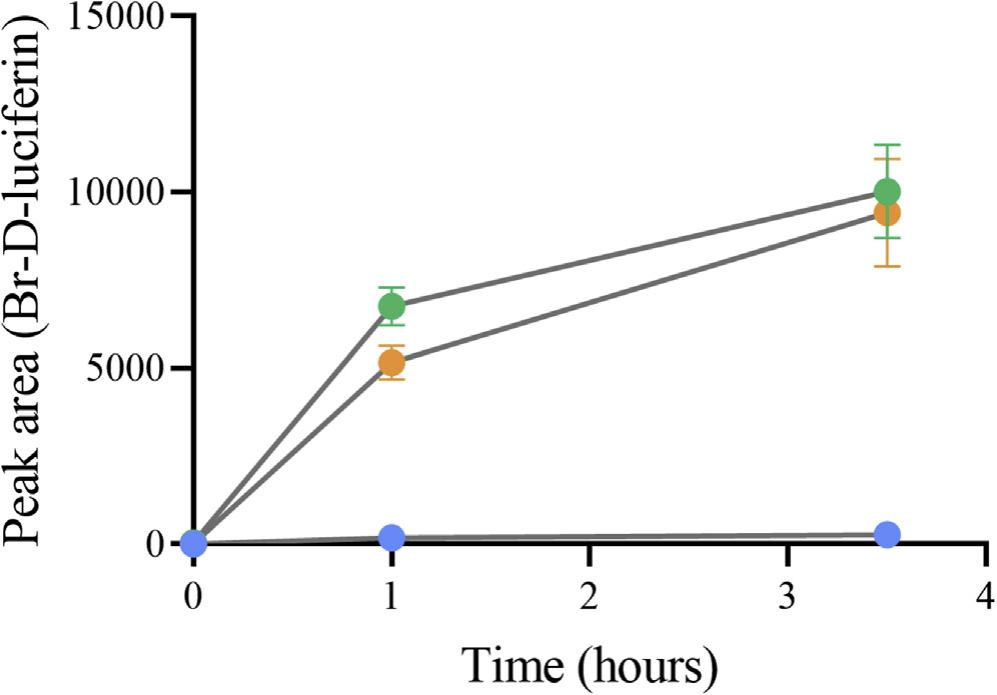
**Comparing****D****-luciferin bromination acitivity of Lys79Thr with wild-type Thal.** Peak areas of brominated D-luciferin product of Lys79Thr (*orange*), wild-type (*green*), and the reaction without Thal (*blue*).

**Figure 8:**
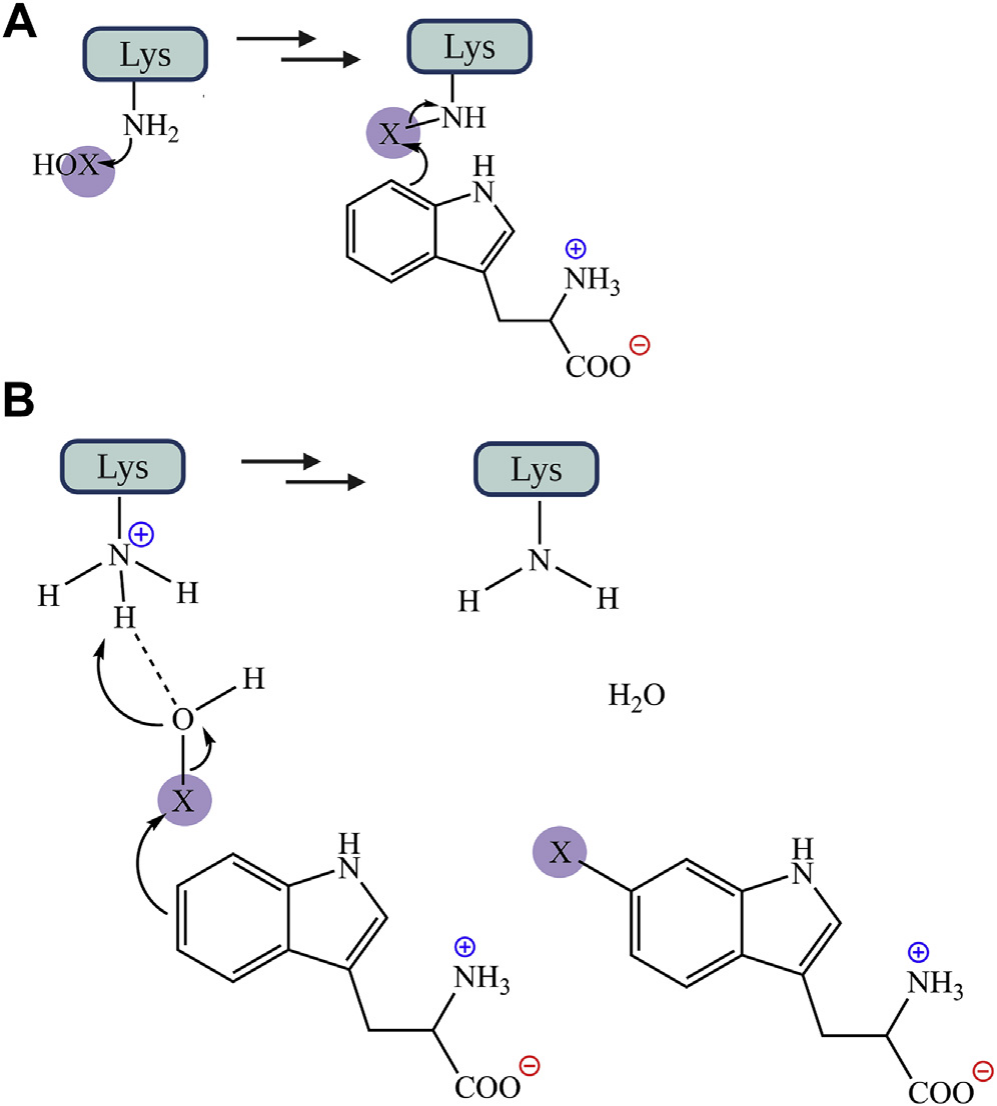
**Proposed reaction mechanisms of tryptophan halogenation.** The reaction was proposed to occur *via* electrophilic aromatic substitution. *A*, in tryptophan 7-halogenase (RebH), the Walsh group proposed that tryptophan can be halogenated by a covalent intermediate (Lys-NH-X) (27). *B*, alternatively, HOX can directly interact with a fully protonated Lys *via* hydrogen bonding (29, 32) to halogenate tryptophan. X represents I, Br, and Cl. Our findings support mechanism B and suggest the role of Lys in protonation of HOX. HOX, hypohalous acid.

Because the catalytic Lys has been proposed to react with HOX (15, 27) to form a chloramine or bromamine intermediate, we investigated if this model is true by measuring the amount of HOX leakage in Lys79Thr compared with the wild-type enzyme (Experimental Procedures). Leakage of HOX was investigated using the HOBr reaction with D-luciferin. Results in Figure 7 showed that the Lys79Thr variant did not give any higher yield of bromo-D-luciferin (higher amount of HOBr) than the wild-type enzyme. The incapability of Lys79Thr to halogenate tryptophan is thus not because of the leakage of HOBr, as it generates a similar level of HOBr as the wild-type enzyme. The data suggest that Lys79 probably does not form a covalent adduct with HOBr or HOCl to form the bromamine or chloramine intermediate as it has been proposed (Fig. 8*A*). Should this have been the case, the Lys79Thr variant would have produced a higher amount of free HOBr to react with D-luciferin.

#### Computational calculations to investigate the pKa of Lys79

As our results in the previous section imply that Lys79 does not form a chloramine or bromamine intermediate, this suggests that their role may be to form hydrogen bonding with HOX as originally proposed by van Pée *et al.* (29). However, the participation of Lys79 in the reaction of Thal is crucial because results from site-saturation mutagenesis experiments showed that Lys79 cannot be replaced by any other amino acids. To investigate why all FDHs contain a strictly conserved Lys as a catalytic residue, propka 3.0 (49) was used for calculating the p*K*a of Lys residues of Thal (PDB code 7CU1) at pH 7.2. The calculation results showed that the p*K*a values of Lys79 in both subunits are abnormally lower, 7.48 (Chain A) and 7.59 (Chain B), than other Lys residues which have typical p*K*a values around 9.0 to 11.0 (Table S4). This p*K*a value allows Lys79 to be in the fully protonated state and can also act as a proton donor during turnovers (Fig. 8*B*). Although Arg has a similar chain length compared with Lys, its higher p*K*a compared with Lys would not promote it to act as a proton donor. Therefore, our study implies that the conserved Lys in FDHs may participate in the reaction by providing a proton for HOX to increase the electrophilicity of X (Fig. 8*B*). Our data do not support formation of a covalent intermediate (chloramine) which was previously proposed for the RebH reaction (27). Results reported here agree with the model proposed in the theoretical study of PrnA (32) and provides more insights into the functional roles of the conserved Lys.

### Mechanisms of Thal:FADH^-^^∗^ inactive complex formation

#### Kinetics of Thal:FADH^-^^∗^ inactive complex formation

**Figure 9:**
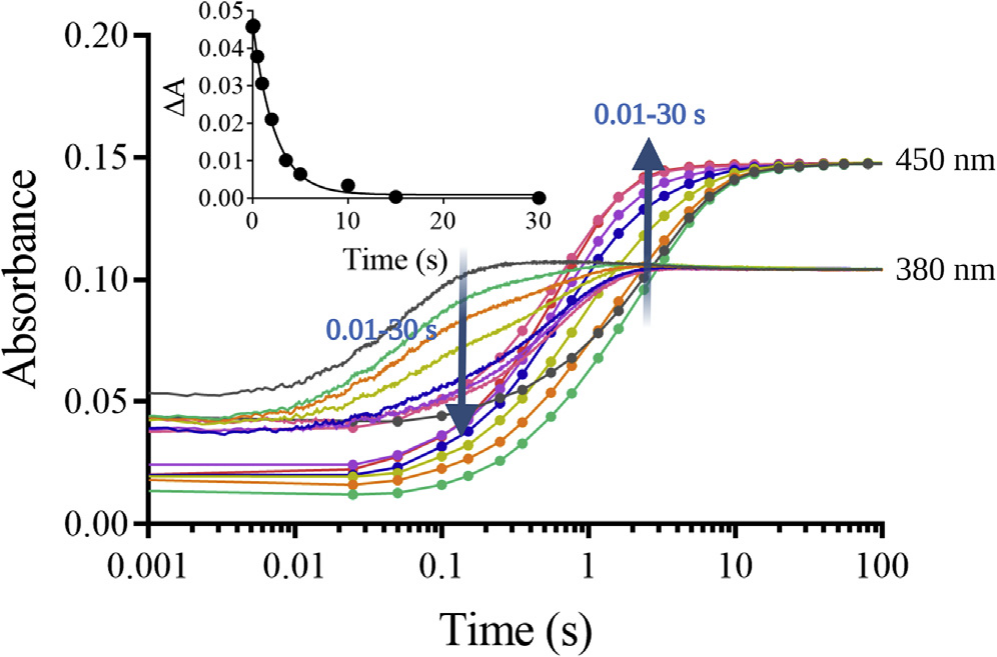
**Kinetics of Thal:FADH^-^^∗^ inactive complex formation.** An anaerobic solution of FADH^−^ was premixed with an anaerobic solution of Thal at various age times (incubation times) (0.01–30 s) in the first mixing. Aerobic buffer was then added into the second mixing step. Final concentrations were 128 μM oxygen, 15 μM FADH^−^, and 30 μM Thal. Kinetic traces were monitored at 380 nm (*solid lines*) and 450 nm (*solid lines with circles*). The inset was a plot of absorbance decrease at 380 nm *versus* incubation time. FADH^−^, reduced flavin adenine dinucleotide.

FDHs are unusal among all two-component flavin-dependent monooxygenases because premixing of the enzymes with FADH^−^ can result in formation of a flavin inactive complex in which C4aOOH-FAD is not detected. This phenomenon was first reported for the RebH reaction (28), but the mechanism causing it has never been investigated. Thal also has this property because C4aOOH-FAD could not be detected from the reaction of mixing a preformed complex of Thal:FADH^−^ with oxygen, unlike the reaction in which a solution of FADH^−^ was freshly mixed with an aerobic solution of Thal (Fig. S5). This must be due to isomerization to form an inactive complex (represented as Thal:FADH^-^^∗^, see more explanation in the structure section) (Fig. 3). Therefore, the kinetics of Thal:FADH^−^ isomerization to form an inactive complex was investigated by double-mixing stopped-flow spectrophotometry. In this experiment, the first mixing setup mixed an anaerobic solution of Thal with an anaerobic solution of FADH^−^ and was incubated over various periods. In the second mixing, an aerobic solution of buffer was added. The amount of C4aOOH-FAD formed could be quantified by the increase in absorbance at 380 nm during the first phase (0.02–0.13 s) as shown in Figure 9.

The results in Figure 9 indicate that the amount of absorbance increase at 380 nm during the first phase diminished upon prolonged incubation. These data indicate that a binary complex of active Thal:FADH^−^ changes over time to form an inactive complex, Thal:FADH^-^^∗^, which does not allow formation of C4aOOH-FAD (Fig. 3). A plot of the decrease in absorbance at 380 nm *versus* incubation time represents the kinetics of the formation of the inactive complex (Fig. 9). The data can be fitted with a single exponential decay (inset of Fig. 9), which corresponds to a rate of isomerization to form an inactive complex as 0.41 s^−1^. Results in Figure 9 showed that the shortest preincubation (0.01 s) gave the highest yield of C4aOOH-FAD formation.

#### The crystal structures of Thal:FADH^−^ and Thal:FAD:AMP complexes explain the mechanism of inactive complex formation

To understand how the inactive complex forms upon prolonged incubation of Thal and FADH^−^, we solved new crystal structures of Thal with bound FADH^−^ or FAD. The structures of Thal:FADH^−^ (PDB code 7CU2) and Thal:FAD:AMP (PDB code 7CU1) were solved with two protomers per asymmetric unit (asu). The overall architecture of Thal is generally very similar to other FDHs. Details of data collection and refinement are shown in Table 3. Clear electron density for FADH^−^ was observed in both protomers. The density of the isoalloxazine ring was not planar, confirming that it is a FADH^−^ not FAD (Fig. S19).

**Table 3:** Data collection and refinement statistics for Thal structures

Parameters	Thal:Trp	Thal:FAD:AMP	Thal:FADH^−^
Data Collection			
Wavelength (Å)	1.54	1.54	1.54
Space group	*P*2_1_	*P*2_1_	*P*2_1_
Cell dimensions			
*a, b, c* (Å)	54.9, 118.3, 87.0	53.6, 118.2, 82.9	54.5, 119.3, 86.6
β (°)	104.4	100.9	104.4
Resolution (Å)	20.75–1.95 (2.05–1.95)	20.66–1.91 (2.01–1.91)	20.96–2.40 (2.50–2.40)
Total reflections	416,775	547,492	223,245
Unique reflections	76,620	77,293	41,355
Completeness (%)	97.9 (97.7)	99.0 (94.1)	98.6 (99.2)
Redundancy	4.0 (2.6)	7.0 (4.4)	4.9 (3.7)
<*l*/*σ*(*l*)>	18.0 (6.8)	17.1 (4.5)	16.7 (6.5)
*R*_merge_ (%)	5.3 (12.2)	7.3 (32.1)	6.6 (16.2)
Refinement			
*R*_*f*_/*R*_*free*_(%)	19.16/24.87	17.44/22.04	20.74/25.89
No. atoms			
Protein	8470	8375 and 63 (tag)	8309
Ligand	30	76	106
Water	855	731	395
*B*-factors (Å^2^)			
Protein	15.21	18.01	23.59
Ligand	12.09	23.87	19.64
Water	19.51	24.27	20.78
Average *B*-factor	15.59	18.56	23.42
Ramachandran plot (%)			
Favored	89.0	90.7	89.5
Allowed	10.9	9.1	10.1
Outliers	0.1	0.1	0.3
R.m.s. deviations			
Bond length (Å)	0.012	0.009	0.011
Bond angle (°)	1.44	1.36	1.48
PDB code	7CU0	7CU1	7CU2

FAD, oxidized flavin adenine dinucleotide; FADH^−^, reduced flavin adenine dinucleotide.

**Figure 10:**
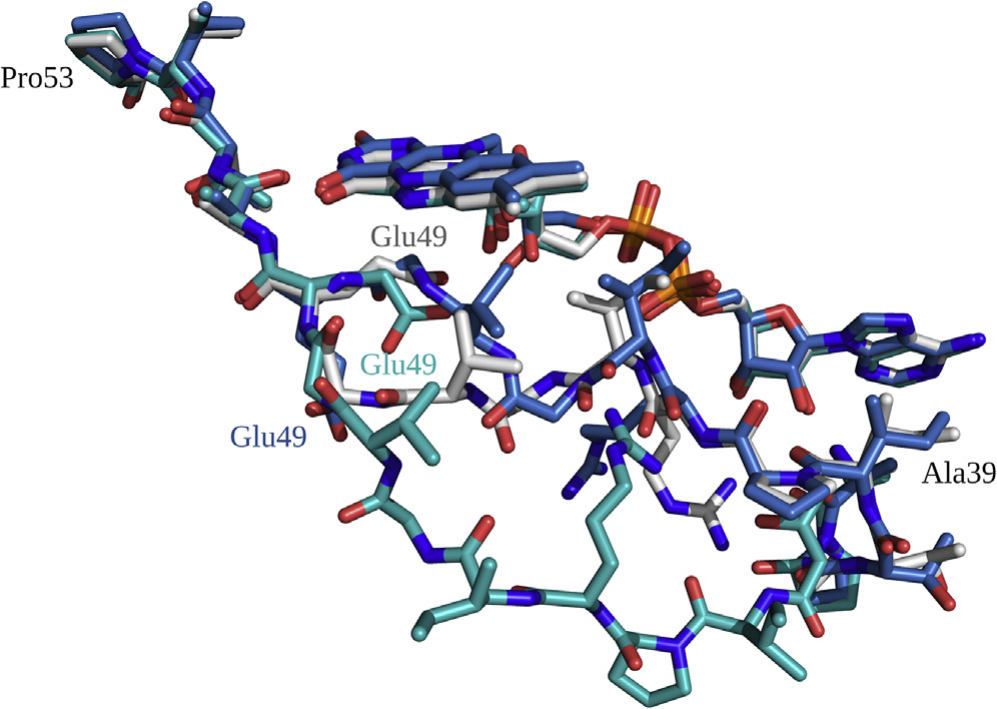
**Alignment of various conformations of the flavin binding loop of Thal structures.** Superposition of conformations of Chain A (*green*) and Chain B (*white*) observed in the Thal:FADH^−^ complex (PDB code 7CU2) defined as “loose” and “tight” conformations, respectively, and Chain A (*blue*) of Thal:FAD:AMP (PDB code 7CU1) defined as “open” conformation. FAD, oxidized flavin adenine dinucleotide; FADH^−^, reduced flavin adenine dinucleotide.

**Figure 11:**
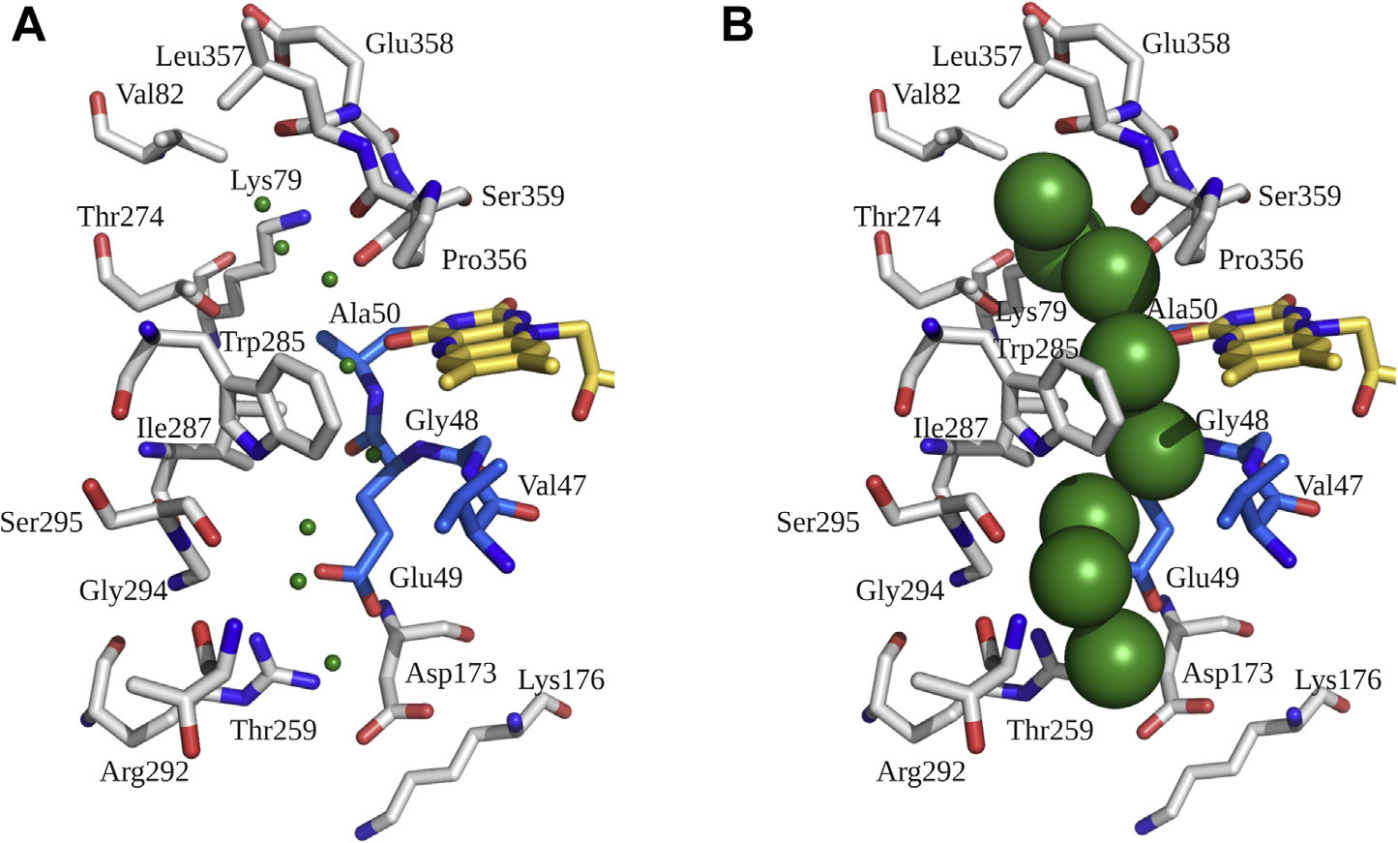
**Water networks observed in the open conformation of Chain A of Thal:FAD:AMP (PDB code 7CU1).***A*, residues within 4 Å of water networks (*green sphere*). *B*, spherical display of water molecules shows water networks connecting bulk solvent to the FAD binding site. FAD, oxidized flavin adenine dinucleotide.

Alignment of Thal:FADH^−^ with Thal:FAD showed different conformations of Thal, especially at the loop around residues 39 to 53 (defined as the flavin binding loop) which are close to the flavin binding site (Fig. 10). Heterogeneity of the flavin binding loop conformation was found, shown as “loose conformation” observed in Chain A, “tight conformation” observed in Chain B of the Thal:FADH^−^ complex, and “open conformation” observed in Chain A of the Thal:FAD:AMP complex (more details and description are provided in Fig. S21). Analysis of these structures indicates that different conformations accommodate different arrangements of water molecules in the active site. Interestingly, in the open conformation, the arrangement of water molecules connects the active site of Thal with the bulk solvent outside (Fig. 11).

**Figure 12:**
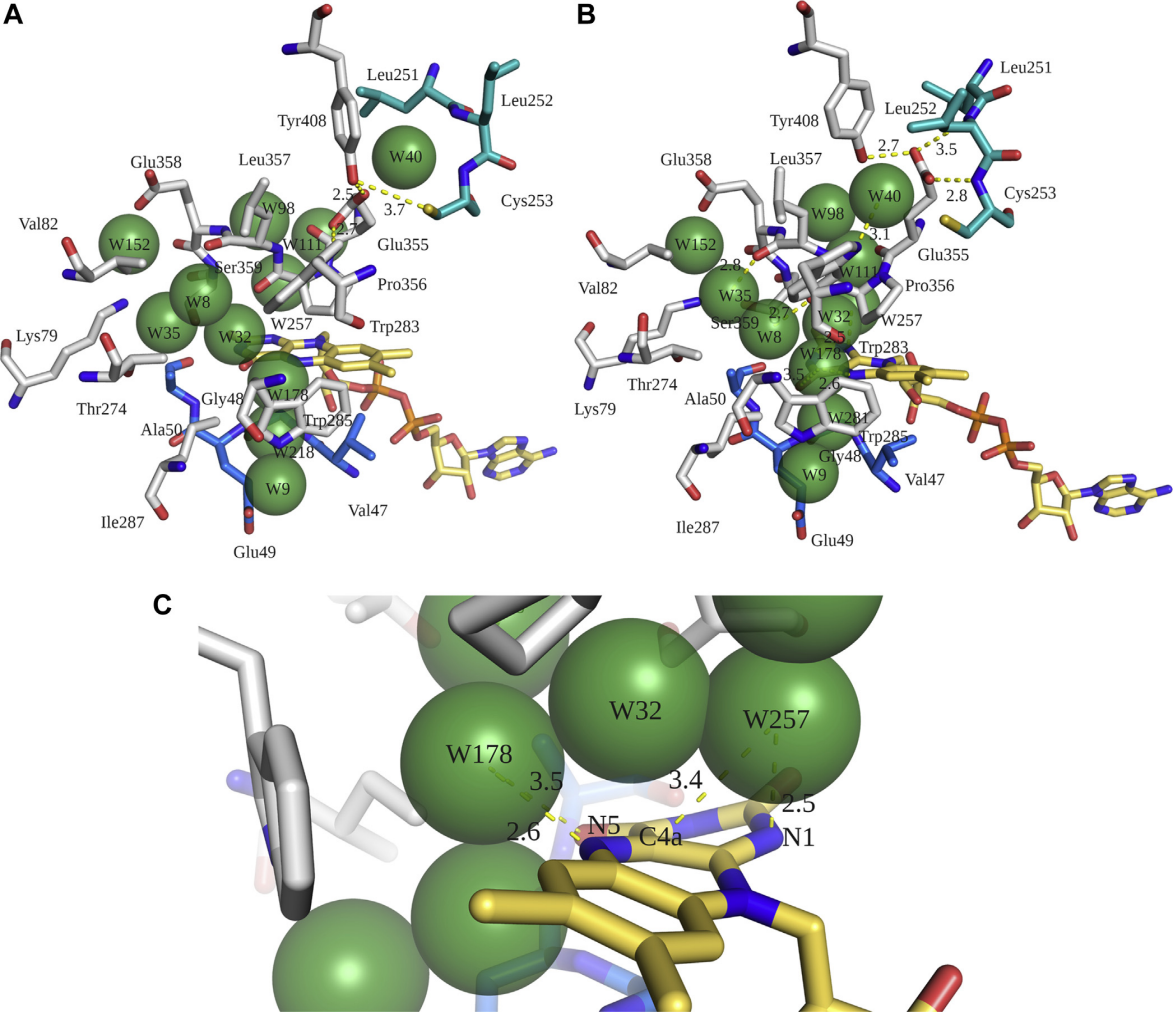
**Rearrangement of water networks resulting from protein dynamic changes.***A*, water network arrangements around the flavin site in Chain A of the Thal:FAD:AMP complex (PDB code 7CU1) were analyzed by MD simulations. Arrangement of water networks observed in the structure of the Thal:FAD:AMP complex (PDB code 7CU1). *B*, rearrangement of water molecules and the *green* loop after 4 ns of the production phase performed by MD simulation. *C*, close-up views of interactions of water molecules around the isoalloxazine ring after 4 ns of the production phase by MD simulations. MD, molecular dynamics; FAD, oxidized flavin adenine dinucleotide.

To elucidate how protein dynamics can affect water arrangement in the active site of Thal, the open conformation was selected as a starting model for MD simulations. For the starting open conformation structure, Glu355 interacts with Cys253, Trp283, Tyr408, and W40 (water molecule) which also interacts with the amide backbones of Leu252 and Cys253 (Fig. 12*A*). No water molecule is located above the C4a position of the isoalloxazine ring. After 4 ns of production phase in MD simulations, Glu355 displaces W40 and interacts with amide backbones of Leu252 and Cys253, resulting in a conformational change in the green loop (Fig. 12, *A*–*B*). Consequently, water molecules rearrange themselves. W35, W8, and W178 form hydrogen bonds with a carbonyl group of Leu357, a carbonyl group of Pro356, and N5 of the isoalloxazine ring, respectively (Fig. 12*B*). Notably, W32 is now located close to the C4a position of the isoalloxazine ring, whereas W32, W178, and W257 are located above the N1 of FAD (2.5 Å) (Fig. 12*C*). Therefore, it can clearly be seen that the conformation of Thal is characterized by significant protein dynamics which in turn affects the arrangement of water molecules in the active site.

**Figure 13:**
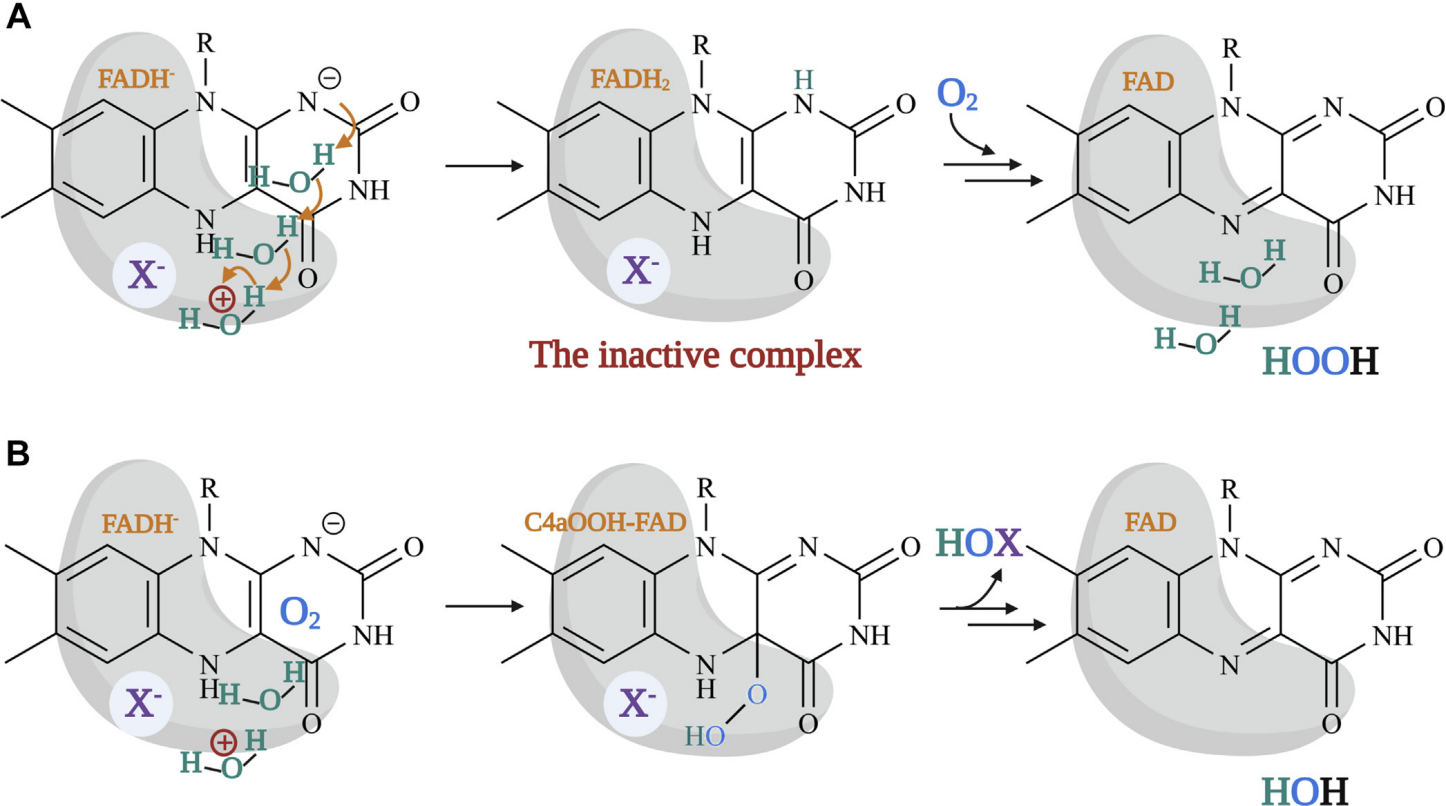
**Mechanism of inactive complex formation in FDHs.***A*, water networks located around the flavin were identified in the crystal structure of Thal. Preincubation of Thal with FADH^−^ under anaerobic conditions promotes conformational changes resulting in water rearrangement around the flavin. These water molecules may promote proton transfer to N1 of FADH^−^ to produce FADH_2_ which is less reactive with O_2_ to form C4aOOH-FAD (inactive complex). *B*, freshly mixing Thal with FADH^−^ under aerobic conditions can avoid inactive complex formation. C4aOOH-FAD, C4a-hydroperoxyflavin; FAD, oxidized flavin adenine dinucleotide; FDHs, flavin-dependent halogenases; FADH^−^, reduced flavin adenine dinucleotide; HOX, hypohalous acid.

Previous work by the Bruice and Massey groups indicated that the deprotonated form of reduced flavin (FADH^−^ not FADH_2_ in the case of FAD, p*K*a value around 6.7) is the form that is more reactive for the oxygen activation process (50, 51, 52). Investigation using density functional theory calculations also confirm this model because the anionic FADH^−^ is the one suitable to undergo proton-coupled electron transfer (PCET) to generate C4a-hydroperoxyflavin in the reactions of the oxygenase component (C_2_) of *p*-hydroxyphenylacetate 3-hydroxylase and pyranose 2-oxidase (53, 54). As water networks in Thal connect the isoalloxazine ring of the flavin to outside bulk solvents, we propose that these water networks are important for providing a proton for the PCET process to stabilize formation of C4aOOH-FAD in the Thal reaction. Upon prolonged incubation of Thal with FADH^−^, the protein conformational changes and rearrangement of water molecule networks may lead to improper protonation status of FADH^−^ such as either to form FADH_2_ which is less reactive with oxygen or it may disturb a protonation process involved in PCET required for the formation of C4aOOH-FAD (Fig. 13).

### Crystal structures of Thal: Implications for limitation of noniodination activity

Because the data in the HOX formation section indicated that Thal could form HOI, we further investigated whether this enzyme could catalyze iodination of tryptophan. Only chlorinated and brominated but not iodinated tryptophan could be detected (Fig. S8). To explore factors controlling halogenation by Thal, we solved the structure of the Thal:TRP complex (PBD code 7CU0, 2.05–1.95 Å) which has a better resolution compared with the previously reported structure (PDB code 6H44, 2.62–2.55 Å) (35).

Structures of Thal and various FDHs including PrnA and PyrH (15, 37) were overlaid and analyzed. Superposition of PyrH, Thal, and PrnA structures showed that the three residues (Lys79, Glu358, and Ser359 in Thal) are found at the same position in all of these enzymes (Fig. S20). Moreover, the halogenation sites of the substrate are all placed next to the side-chain of Lys79. We noticed that the space between the tryptophan substrate and Lys79 was confined by the surrounding residues in the pocket and were not large enough to accommodate the larger sphere of the iodide species. We modeled HOI into the active site and found that the van der Waals sphere of the tryptophan does not allow this putative HOI to fit (data not shown). The tight space between the halogenation site of tryptophan and the residues lining the binding pockets would not allow HOI to be accommodated. These data clearly explain the results in the HOX formation section which show that although HOI can be formed at the flavin site, iodination of tryptophan cannot occur because of the limited space in the tryptophan binding site.

**Figure 14:**
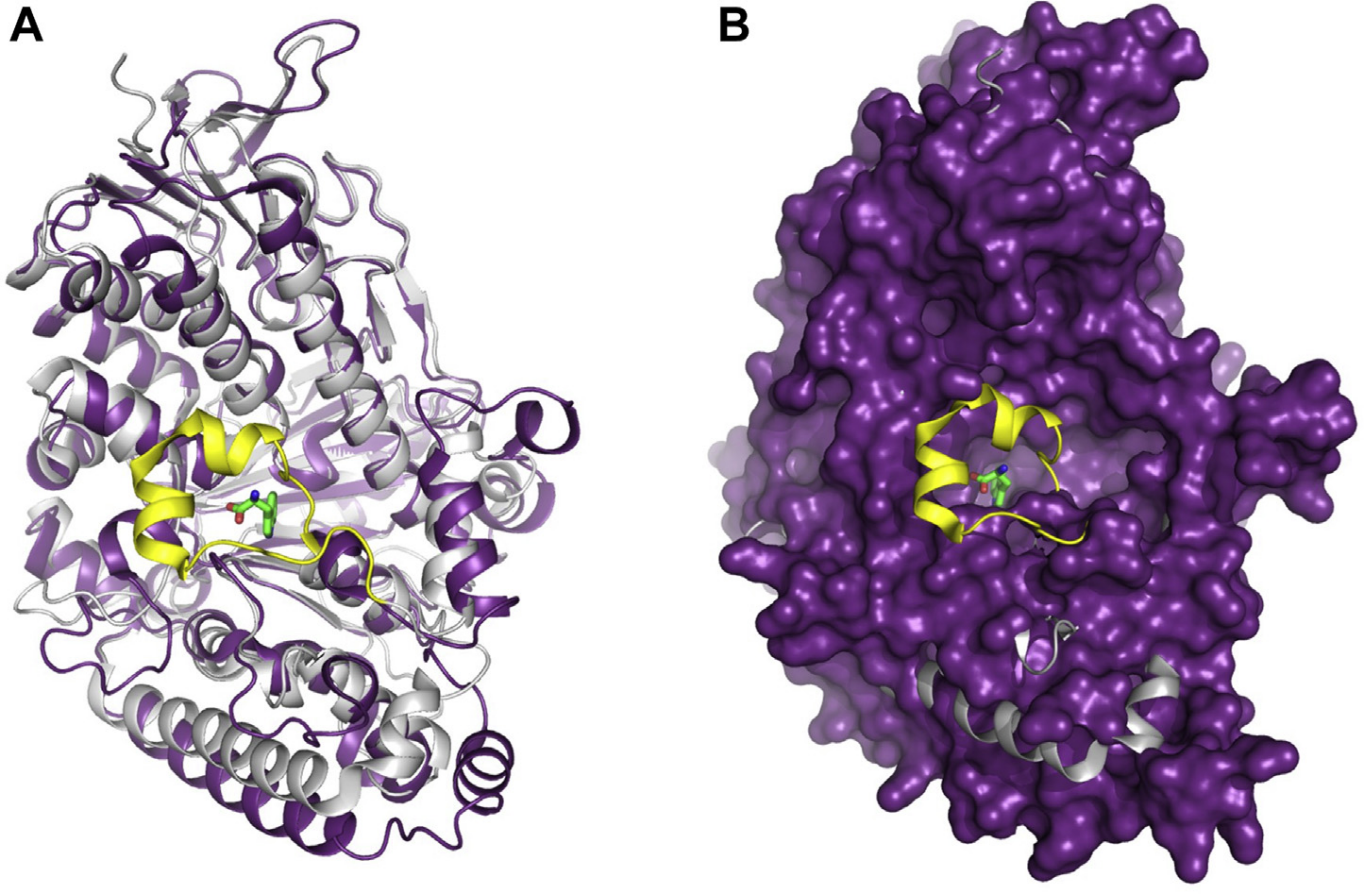
**Comparison of structures of Thal (PDB code 7CU0) and VirX1 (PDB code 6QGM).***A*, alignment of VirX1 (*purple*) and Thal (*gray*) structures identified the distinct α-helical (*yellow*) structure encompassing the Trp substrate (*green stick*) in Thal. *B*, surface representation of VirX1 aligned with Thal.

Recently, the viral FDH (VirX1) was identified, and it was reported that it can use various halides, including I^−^, Br^−^, and Cl^−^ (31). It showed iodination activity toward a 6-azaindole substrate (31). VirX1 has 29% sequence similarity to PrnA (8, 17). Structures of VirX1 with Thal (PDB code 7CU0) are similar especially at the FAD binding site. Although the structure of VirX1 is similar to Thal, VirX1 has a higher degree of substrate flexibility and halide utilization. A key difference between VirX1 and Thal is found in the helical lid (residue 445–466) (Fig. 14). The absence of an α-helical lid in VirX1 results in a larger substrate pocket, allowing VirX1 to use a broad range of substrates and halides including I^−^. Thus, the helical lid is the key factor preventing Thal catalysis of tryptophan iodination.

## Discussion

Our results reported herein highlight four new key mechanistic insights on FDHs: (A) C4aOOH-FAD formed by tryptophan halogenases can react with I^−^, Br^−^, and Cl^−^ to form HOI, HOBr, and HOCl, respectively. However, iodination of tryptophan cannot occur because there is not enough space to accommodate the HOI in the tryptophan binding site. (B) Using D-luciferin for detection of free HOX, we observed leakage of HOX from Thal, and this feature probably contributes to the low product coupling ratio of the enzyme. (C) Site-saturation mutagenesis showed that the active site Lys is indeed crucial because none of the other 19 amino acids can replace the function of Lys. Computational results indicate that the active site Lys has an abnormally lower p*K*a compared with other Lys residues, possibly allowing it to act as a proton donor for HOX during halogenation. (D) Stopped-flow experiments have shown that prolonged incubation of Thal with FADH^−^ leads to formation of an inactive enzyme species in which C4aOOH-FAD cannot be detected. Various conformations of newly solved Thal:FADH^−^ (PDB code 7CU2) and Thal:FAD:AMP (PDB code 7CU1) structures combined with MD simulations revealed that protein conformational changes and water molecule arrangement may lead to inactive complex formation.

Our stopped-flow results clearly showed that all tryptophan halognases (Thal, PrnA, and PyrH) can react with all halide ions (I^−^, Br^−^, and Cl^−^) except F^−^ to yield the C4aOH-FAD intermediate and a halogenating species, *i.e.*, HOX. Rate constants of the formation of the halogenating species showed that I^−^ gave the highest rate constant. These data are well correlated with the QM/MM calculations, indicating that the potential energy barrier to form HOX is the lowest for the reaction of C4aOOH-FAD with I^−^. Although HOI can be formed at the flavin site, an iodinated tryptophan could not be detected as a product. This is likely because of the constrained space in the tryptophan binding site that does not accommodate the larger iodide species.

The site-saturation mutagenesis and computational studies reported here can explain the mechanistic role of the catalytic Lys. Although all FDHs discovered to date have a strictly conserved Lys at their active sites, it is still unclear whether the residue can form a chloramine intermediate (27) or hydrogen bonds with HOX (29, 32). Only site-directed mutation to Ala (15, 27) or Arg (31) were investigated in the past. Our site-saturation mutagenesis results show that all variants are inactive, indicating that the catalytic Lys is not dispensible. We found that the catalytic Lys is unique for its abnormal p*K*a value and has the lowest p*K*a compared with other Lys residues. We propose that this catalytic Lys may serve as a proton donor to HOX (Fig. 8*B*). Our findings agree with the mechanism previously proposed that Lys does not form a chloramine intermediate (Fig. 8*B*) (29, 32).

Kinetics and crystal structures of Thal have also shown that the Thal structure is highly dynamic. Upon prolonged incubation, the Thal:FADH^−^ complex can isomerize to an inactive form which C4aOOH-FAD cannot be detected (Figs. S5 and S6). This is similar to the property of RebH which could also form the inactive complex after prolonged incubation (28). Similar phenomena were also found in several two-component flavin-dependent monooxygenases. A dechlorinating/denitrating flavin-dependent monooxygenase (HadA) from *R. pickettii* DTP0062 also shifts to form a species which is less reactive with oxygen upon longer preincubation of FADH^−^ and HadA (38). FMNH^−^-dependent alkanesulfonate monooxygenase (SsuD) from *E. coli* has also been reported to form an inactive complex when SsuD is preincubated with FMNH^−^ in the absence of oxygen (55). However, some flavin-dependent monooxygenases, *i.e.*, the monooxygenase component (C_2_) of *p*-hydroxyphenylacetate hydroxylase, does not have any problem when the enzyme is preincubated with FMNH^−^ (39). As shown in Figure 10, Figure 11, Figure 12, Figure 13 and mentioned in the Results section, Thal protein conformational changes can cause water rearrangements which may lead to an improper protonation status of FADH^−^ or disrupt an arrangement which facilitates the PCET process for C4aOOH-FAD formation. It is interesting to note that enzymes which are robust in formation of C4aOOH-flavin such as C_2_ use an active site residue (His396 in C_2_) not water networks as a proton donor for the PCET process. We speculate that this arrangement can be more precise in controlling the protonation status of reduced flavin and the PCET process than Thal which relies on water networks to transfer protons.

In conclusion, our data have expanded the understanding of the mechanism of halide ion reactivity of tryptophan halogenases. Although tryptophan halogenases can form reactive halogenating species, HOI, HOBr, and HOCl, the enzyme can only catalyze chlorination and bromination but not iodination because the space at the tryptophan binding site cannot accommodate the halogenating iodine species. We believe that future efforts to widen the substrate binding site may allow some tryptophan halogenases such as Thal to catalyze the iodination reaction. We also found HOX leakage which might be the cause for the low catalytic inefficiency of FDHs in general. Future efforts in enzyme engineering to decrease HOX leakage may improve the halogenation activity of FDHs. The catalytic Lys has an abnormally low p*K*a which allows it to serve as a proton donor during product formation. The fundamental catalytic properties reported should be useful for improvement of halogenases to serve as green biocatalysts in the future.

## Experimental procedures

### Single turnover reaction of Thal

Single turnover of tryptophan bromination activity of Thal was carried out in a Hi-Tech Scientific Model RQF-63 rapid-quench flow apparatus at 25 °C. An air-saturated solution containing Thal, NaBr, and D,L-tryptophan in 20 mM potassium dihydrogen phosphate buffer pH 7.2 was mixed with an anaerobic solution of FADH^−^, and the reactions were stopped at different time points by quenching the reaction with 0.4 M HCl. Final concentrations after quenching were 15 μM Thal, 7.5 μM FADH^−^, 15 μM D,L-tryptophan, and 5 mM NaBr. Reactions were analyzed by HPLC-MS/MS using an Eclipse Plus C18 column (3.5 μm, 4.6 × 100 mm) with a gradient mobile phase of 95:5 to 20:80 of H_2_O:acetonitrile containing 0.1% (v/v) formic acid as an eluent at a flow rate of 0.5 ml/min and monitoring for mass/charge (m/z) of tryptophan and bromotryptophan. Multiple reaction monitoring mode of HPLC-MS/MS with positive ion mode was set for the dectection.

### Transient kinetics experiments investigated by stopped-flow spectrometry

Rapid kinetics experiments were performed using a TgK Scientific model SF-61DX stopped-flow in single- or double-mixing mode or with a model SF-61SX stopped-flow spectrophotometer in single-mixing mode. An oxygen-scrubbing solution, as previously described (39), was used for scrubbing trace amounts of oxygen from the apparatus to make the system anaerobic. Reactions were carried out in 20 mM potassium dihydrogen phosphate buffer, pH 7.2, 25 °C. Trace amounts of oxygen were removed from the glass tonometer by evacuating air and purging the system with nitrogen gas. FAD was reduced in a glove box (Belle Technology, Weymouth, UK) to obtain FADH^−^ by titration with sodium dithionite. Flavin intermediates were monitored by UV-Visible or fluorescence spectrometry. Apparent rate constants (*k*_obs_) were analyzed using Program A software (written by R. Chang, J. Chiu, J. Dinverno, and D. P. Ballou, University of Michigan). More details for each experiment are described in Supplemental Information.

### QM/MM calculations

The reactivity of C4aOOH-FAD with different halide ions to form C4aOH-FAD and HOX was investigated using QM/MM. The crystal structure of the Thal:FAD:AMP complex (PDB code 7CU1) was used for these calculations. The model setup and the QM/MM calculations are described in the Supporting Information. We applied protocols similar to those used previously in QM/MM investigations of other enzyme-catalyzed reaction mechanisms (43, 44). The positions of hydrogen atoms in the enzyme were located using the CHARMM procedure HBUILD (56). The protonation states of titratable amino acid residues were chosen based on predictions from PROPKA (57). The atom types in the topology files were assigned based on the CHARMM27 parameter set (58). The system was divided into QM and MM parts for QM/MM simulations. The QM part consisted of Cl^−^ and C4aOOH-FAD with a net charge of 0 e. The model was divided into the reaction zone which is an area of 21 Å radius, centered on the carbon atom C4a of C4aOOH-FAD molecule, and the buffer zone is the rest of the protein system. First, the energy system was minimized using Adopted Basis Newton-Raphson minimization with the AM1/CHARMM27 QM/MM method. The system was then equilibrated with QM/MM molecular dynamics at the AM1/CHARMM27 level. MP2/6-31G(d),I=lanl2dz/CHARMM27 calculations on the reaction (59, 60, 61) were performed with the QoMMMa program (62). The optimized structure from this *ab initio* QM/MM reaction modeling calculations was used for higher level *ab initio* QM/MM single point calculations using the Molpro program (63). Energy profiles were calculated at the SCS-MP2/aug-cc-pvdz,I=auc-cc-pwcvdz-pp/CHARMM27 level; the SCS-MP2 method has been shown to give accurate results, close to coupled cluster calculations for other enzyme-catalyzed reactions (43, 44, 45, 46). More details of the calculations are given in the Supporting Information.

### Investigation of HOX leakage from Thal

Experiments were done by performing multiple turnover reactions in the absence of substrate tryptophan to produce HOX. In this study, HOBr was generated after 1.5 h of incubation with 20 mM potassium dihydrogen phosphate buffer pH 7.2, 20 μM FAD, 20 μM NAD^+^, 100 mM NaBr, 0.4 μM flavin reductase (C_1_), 25 mM HCOONa, 0.2 μM formate dehydrogenase from *Pseudomonas* sp. (psFDH), and 20 μM Thal at room temperature (22–25 °C). Enzymes in the reaction were removed by centrifugation through Microcon (10 kDa cut-off). D-luciferin (2 μM final concentration) was added into the reaction solution. After incubation for 2 h, reactions were analyzed using HPLC-diode array detector/MS and HPLC-MS/MS.

### Characterization of brominated D-luciferin products using HPLC-QTOF

A large scale reaction (6.3 ml) of D-luciferin bromination was set up to characterize the products. The reaction was comprised of 20 μM FAD, 20 μM NAD^+^, 100 mM NaBr, 0.3 μM C_1_, 25 mM HCOONa, 0.2 μM psFDH, 5 μM D-luciferin, and 20 μM Thal (wild-type) in 20 mM potassium dihydrogen phosphate buffer pH 7.2. Reactions were incubated for 48 h at room temperature (22–25 °C) in a dark room. Fresh enzymes including Thal, C_1_, and psFDH were added into the reaction mixtures every 4 h. Enzymes in the reaction were removed by centrifugation through a Microcon (10 kDa cut-off). Reactions were analyzed using HPLC-QTOF.

### Site-saturation mutagenesis of Thal at Lys79

For the site-saturation mutagenesis at the Lys79 position, NNK-degenerate oligonucleotides were designed as showed in Table S2. Using PCR amplification, *Thal* (wild-type) containing GFP11 tag in the pET28b vector was used as a template (47). PCR products were purified from an agarose gel, and the products were treated with DpnI and cutsmart buffer at 37 °C for 16 h to remove the *Thal* (wild-type)-pET28b plasmid. Plasmids were transformed into BL21(DE3) competent cells by the heat shock method. LB broth (900 μl) was added to transformed BL21(DE3) cells. Cell cultures were grown at 37 °C and 220 rpm for 2 h before centrifugation and discarding 900 μl of supernatant. The remaining 100 μl were resuspended and spreaded onto 15 ml of LB agar containing 25 μg/ml kanamycin. Cultures were further grown at 37 °C for 16 h. Cells were harvested by resuspending colonies using 3 ml of LB, and extracted DNA was used for sequence analysis to evaluate site-saturation libraries (64). Libraries which had Q_pool_ values of at least 0.7 were further cultured on LB agar plates containing 50 μg/ml kanamycin for 16 h. A total of 168 colonies of Lys79 variants, 12 colonies of wild-type, six colonies of BL21(DE3) as negative controls, respectively, were cultured in 96-well plates containing TB medium with and without kanamycin. Cells were cultured at 37 °C with shaking at 50 rpm for 16 h. Cells were divided into two parts. The first part was kept as a library stock by mixing each cell cuture with glycerol to make up a final concentration of 25% (v/v) and stored at −80 °C. The second part was used for assay of protein level and tryptophan bromination.

Gene expression was done by transferring 7 μl of a starter culture into 200 μl of TB medium (3% v/v inoculation). Cells were cultured at 37 °C with shaking at 50 rpm for 7 h. Then, isopropyl β-D-1-thiogalactopyranoside with a final concentration of 1 mM was added into the culture, and the temperature was switched from 37 to 25 °C. Cells were further cultured at 25 °C with shaking at 50 rpm for 16 h. Cells were harvested and lysed. Lysates were centrifuged for 30 min at 4000 rpm 4 °C. The supernatants were taken for activity assay and protein level analysis.

Gene expression yields were determined using high throughput GFP split system (47, 48). Thal was tagged with a small portion of GFP sequence called a GFP11 at the C-terminal (Thal-GFP11). The purified GFP1-10 (150 μl) was incubated with 20 μl of supernatant at 4 °C for 1 day. The protein levels were determined by measuring fluorescence intensity at an emission wavelength of 530 nm using an excitation wavelength of 488 nm which resulted from the interaction of GFP1-10 and Thal-GFP11.

The supernatant (40 μl) was transferred to new 96-well plates. A solution of 12.5 mM NaBr, 10 μM NAD^+^, 10 μM FAD, 22 mM HCOONa, 0.1 mM tryptophan, and 0.1 μM C_1_ in 20 mM potassium dihydrogen phosphate buffer pH 7.2 was added (50 μl) into each well. The reaction was initiated by the addition of 0.165 μM psFDH to each well, and the reactions were incubated at 60 rpm, 25 °C. After the reactions were incubated for 3 h, they were quenched with 0.2 M HCl and analyzed using HPLC-MS/MS with the same conditions as described in the single turnover reaction section.

### Crystallization of Thal

Crystals of Thal apoenzyme and tryptophan complexes were obtained from a microbatch crystallization (65) in a solution containing 0.28 to 0.38 M NaF, 23% to 31% (w/v) PEG3350. Thal:FAD:AMP and Thal:TRP crystals were obtained from soaking of apo Thal crystals into a well containing crystallizing solution and FAD and Trp at a ratio of 2.2:5 mM for 7 days and 0.8:1.6 mM for 2 days, respectively at 25 °C. Thal:FADH^−^ co-complex crystals were produced by placing Thal:Trp complex crystals in a well containing crystallizing solution containing 5 mM tryptophan, 4 mM FAD, and 20% (v/v) glycerol at 25 °C followed by the addition of Na_2_S_2_O_4_ powder. The crystals were harvested by flash-freezing in liquid nitrogen. Diffraction data were collected from the flash frozen crystals on a single crystal X-ray diffraction D8 venture at the Characterization and Testing Center, National Science and Technology Development Agency, Thailand. Data processing was performed with Proteum3 software. Structural phases were solved by molecular replacement using PHASER (66) in the CCP4 suite (67) with SttH (PDB code 5HY5) as a search model. Structure refinement and building were performed with REFMAC5 (68) and COOT (69, 70). Parameter files of FADH^−^ for refinement was generated with LIBCHECK in the CCP4 suite. The structures were validated in PROCHECK (71). LigPlot (72) and PyMOL (https://pymol.org/) was used for molecular graphics.

Figures were created with ChemBioDraw Ultra (Version 15.1), BioRender.com, and the PyMOL Molecular graphic System (Version 2.3.4).

## Data availability

All data are contained within the manuscript and Supporting Information. Data of X-ray structures are available at Protein Data Bank under PDB codes indicated.

## Conflict of interest

The authors declare that they have no conflict of interest with the contents of this article.
