# Supplementary material for: Dissecting the low catalytic capability of flavin-dependent halogenases
Source: J Biol Chem. 2020 Nov 23;296:100068. doi: 10.1074/jbc.RA120.016004 (PMC7948982; doi:10.1074/jbc.RA120.016004)
Supplement: Supplementary Figure and Tables [file mmc1.pdf]

Dissecting the low catalytic capability of flavin-dependent halogenases

Aisaraphon Phintha[1,2#], Kridsadakorn Prakinee[2#], Aritsara Jaruwat[3], Narin Lawan[4], Surawit Visitsatthawong[2], Chadaporn Kantiwiriyawanitch[2], Warangkhana Songsungthong[3], Duangthip Trisrivirat[2], Pirom Chenprakhon[5], Adrian Mulholland[6], Karl-Heinz van Pée[7], Penchit Chitnumsub[3*], Pimchai Chaiyen[1,2]*

[1]Department of Biochemistry and Center for Excellence in Protein and Enzyme Technology, Faculty of Science, Mahidol University, Bangkok, Thailand, 10400.
[2]School of Biomolecular Science and Engineering, Vidyasirimedhi Institute of Science and Technology (VISTEC), Wangchan Valley, Rayong, Thailand, 21210.
[3]Biomolecular Analysis and Application Research Team, National Center for Genetic Engineering and Biotechnology, 113 Thailand Science Park, Paholyothin Road, Klong 1, Klong Luang, Pathumthani, Thailand, 12120.
[4]Department of Chemistry, Faculty of Science, Chiang Mai University, Chiang Mai, Thailand, 50200.
[5]Institute for Innovative Learning, Mahidol University, Nakhon Pathom, Thailand, 73170.
[6]Centre for Computational Chemistry, School of Chemistry, University of Bristol, Cantock's Close, Bristol BS8 1TS, U.K.
[7]General Biochemistry, Faculty of Chemistry and Food Chemistry, Technical University of Dresden, Dresden, Germany.

*Running title: Mechanisms and Structures of Tryptophan-6-Halogenase

*Corresponding authors
E-mail: pimchai.chaiyen@vistec.ac.th
E-mail: penchit@biotec.or.th
#These authors contributed equally.

Keywords: flavin monooxygenase, halogenase, X-ray structures, kinetics, stopped-flow, QM/MM calculations.

Table of contents

	Page
Construction of tryptophan halogenase genes for gene expression	S-4
Gene expression in *E. coli*	S-4
Protein purification	S-4
Determination of the native molecular mass of Thal	S-5
Tryptophan chlorination activity of tryptophan halogenases	S-6
Transient kinetics experiments investigated by stopped-flow spectrometry	S-10
Product analysis of tryptophan halogenation by Thal	S-14
QM/MM calculations to explain the reactivity of C4aOOH-FAD towards different halide ions	S-15
Site-saturation mutagenesis at position $Lys79^{Thal}$	S-21
Crystallographic ligand density in Thal structures	S-25
Conformation changes of Thal explain formation of inactive complex	S-27
Molecular dynamics (MD) analysis to explain inactive complex formation	S-28
References	S-28
Table S1. Separation of protein markers by FPLC	S-5
Table S2. Primers for construction of Lys79 mutant libraries	S-21
Table S3. Amount of Thal enzyme obtained after purification	S-21
Table S4. p*K*a of Lys residues in Chain A and Chain B of Thal (PDB code 7CU1) calculated by propka3.0	S-22
Figure S1. A standard curve of protein markers for determination of Thal native molecular weight	S-5
Figure S2. Tryptophan chlorination activity of tryptophan halogenases	S-7
Figure S3. Identification of tryptophan chlorination by tryptophan halogenases analyzed by HPLC-DAD-MS (ESI)	S-8
Figure S4. NMR spectra of tryptophan and 6-chlorotryptophan	S-9
Figure S5. Reaction of Thal:$FADH^-$ with oxygen	S-11
Figure S6. Reaction of Thal:$FADH^-$ with oxygen under light-minimization conditions	S-11
Figure S7. Kinetic traces of PrnA and PyrH reactions with various halide ions	S-13

Figure S8. Product analysis of tryptophan halogenation by Thal S-14

Figure S9. Reaction mechanism for the HOX formations catalyzed by flavin-dependent halogenases S-17

Figure S10. Chemical structures of C4aOOH-FAD and Cl^- along the reaction S-18

Figure S11. Chemical structures of C4aOOH-FAD and Br^- along the reaction S-18

Figure S12. Chemical structures of C4aOOH-FAD and I^- along the reaction S-18

Figure S13. SCS-MP2/aug-cc-pvdz/CHARMM27 PES for HOCl formation S-19

Figure S14. SCS-MP2/aug-cc-pvdz/CHARMM27 PES for HOBr formation S-19

Figure S15. SCS-MP2/aug-cc-pvdz,I=auc-cc-pwcvdz-pp/CHARMM27 PES for HOI formation S-20

Figure S16. Mulliken charge analysis for the HOCl formation catalyzed by Thal S-20

Figure S17. DNA sequencing read by fluorescence of site-saturation mutagenesis of a Thal library S-23

Figure S18. Tryptophan bromination activity of Thal wild-type and Lys79Thr variants S-24

Figure S19. Electron density of $FADH^-$ and FAD S25

Figure S20. Tryptophan arrangement for regioselectivity in Thal, PrnA and PyrH S26

Figure S21. Different conformations of the flavin binding loop S27

Construction of tryptophan halogenase genes for gene expression.

Codon optimization and gene synthesis of tryptophan 7-halogenase (*PrnA* (1,617 bp)), tryptophan 5-halogenase (*PyrH* (1,536 bp)), and tryptophan 6-halogenase (*Thal* (1,596 bp)) for expression in *Escherichia coli* (*E. coli*) were performed by GenScript. All genes were digested with NdeI and HindIII restriction enzymes (Novagen) at the 5' and 3' ends, respectively. Each of the genes was ligated into a pET28a vector. Sequence analysis was done by Macrogen.

Gene expression in *E. coli*.

The expression of *thal* and production of Thal were previously done in the *E. coli* system using isopropyl β-D-1-thiogalactopyranoside (IPTG) and L-arabinose induction (1). In this study, we constructed the clone for overexpression of *thal* from *S. albogriseolus* in *E. coli* BL21(DE3) using an auto-induction system. Recombinant plasmids containing the *thal* gene were introduced into BL21(DE3) cells. Cells were grown on LB agar plates containing 50 µg/mL kanamycin at 37 °C for 16 hours. Starter cell cultures were prepared by inoculating a single colony of cells containing the recombinant plasmid into 100 mL of LB medium (ZYM-5052 medium for Thal) containing 50 µg/mL kanamycin at 220 rpm, 37 °C for 16 hours. For growth and expression of *thal*, 1% v/v of starter culture was inoculated into 650 mL ZYP-5052 media containing 50 µg/mL kanamycin. Cells were grown at 220 rpm, 37 °C until OD_{600} reached 1.5. The temperature was then reduced to 25 °C, and the cell culture was continuously grown for 16 hours. Because other tryptophan halogenases (PrnA and PyrH) were also used in this study for investigation of halogenation kinetics, the production of these two enzymes was also carried out in *E. coli* with IPTG induction system. The culture of PyrH (0.5% v/v) or PrnA (1% v/v) was then inoculated into 650 mL of LB media containing 50 µg/mL kanamycin. For growth and expression of *prnA* and *pyrH*, cells were grown at 37 °C with shaking at 220 rpm until the OD_{600} reached 1.5 and the temperature was then switched to 25 °C. IPTG was added at a final concentration of 1 mM. The cells were then grown at 25 °C with shaking at 220 rpm for 16 hours.

Protein purification.

Cell paste was suspended in a lysis buffer which contained 1 mM dithiothreitol (DTT), 100 µM phenylmethylsulfonyl fluoride (PMSF) and 5 mM ethylenediaminetetraacetic acid (EDTA) in 10 mM potassium dihydrogen phosphate buffer pH 7.2. Cells were lysed by an ultrasonication. Cell debris was removed by centrifugation at 28,980 x g for 30 minutes. Polyethylenimine (PEI) 0.5 % (v/v) was used for precipitation of nucleic acids and the resulting precipitation was then removed by centrifugation. The supernatant was dialyzed against 4 liters of 5% (v/v) glycerol in 10 mM potassium dihydrogen phosphate buffer pH 7.2 for overnight. Denatured proteins were removed by centrifugation. The supernatant fraction was loaded onto a nickel (2+) immobilized chelating Sepharose Fast Flow column (GE Healthcare), which was pre-equilibrated with 10 mM potassium dihydrogen phosphate buffer pH 7.2 containing 5% (v/v) glycerol and 150 mM NaCl, buffer A. To remove unwanted proteins, the column was washed with 10 column volumes of buffer A containing imidazole 50 mM (PrnA and PyrH) or 30 mM (Thal). Isocratic buffer A containing imidazole 120 mM (Thal), 150 mM (PrnA) or 200 mM (PyrH) was used for enzyme elution. Fractions containing halogenases were concentrated. Concentrated samples were then exchanged into 10 mM potassium dihydrogen phosphate buffer pH 7.2 containing 20% (v/v) glycerol using a Sephadex G-25 column (GE Healthcare). Purified proteins were stored at -80 °C until used. All purification processes were carried out at 4 °C. Purity of proteins was verified by sodium dodecyl sulfate polyacrylamide gel electrophoresis (SDS-PAGE) analysis. Protein concentration was measured using UV-Vis spectrometry at 280 nm. Extinction coefficients of proteins (PrnA ε_{280} = 97.29 $mM^{-1}cm^{-1}$, PyrH ε_{280} = 98.78 $mM^{-1}cm^{-1}$, and Thal ε_{280} = 87.32 $mM^{-1}cm^{-1}$) were calculated using the ProtParam tool of ExPASy.

Results showed that Thal, PyrH, and PrnA had about 99% purity (data not shown). The purification protocol resulted in 13.3, 169.9 and 185.0 mg of PrnA, PyrH and Thal from 3.9 liters of each culture, respectively.

Determination of the native molecular mass of Thal.

The native molecular mass of Thal was determined by fast protein liquid chromatography (FPLC). Analysis was done using high-resolution gel filtration column (Superdex 200 HR10/300 GL). The samples (500 µL) were injected, then eluted with an isocratic mobile phase of 10 mM potassium dihydrogen phosphate buffer pH 7.2 at a flow rate of 0.75 mL/min. Proteins were detected using a diode array detector at 280 nm. Alcohol dehydrogenase, albumin, carbonic anhydrase and cytochrome C (Sigma) were used as molecular weight markers. Molecular masses, void volumes (V_0), and elution volumes (V_e) of the proteins analyzed are shown in Table S1. The standard curve represents a relationship between the logarithmic values of the molecular weight of known standards and V_e/V_0 as shown in Fig. S1. Thal eluted at V_e = 15.76 mL, corresponding to a native molecular weight of 109,150 Da based on correlation with the standard curve. As the molecular weight of the Thal subunit is 62,183 Da based on amino acid sequence information and SDS-PAGE analysis (data not shown), these data indicate that the quaternary structure of Thal is a dimer.

Table S1

Separation of protein markers by FPLC.

Protein	MW (Da)	Log MW	V_e (mL)	V_0 (mL)	V_e/V_0
Alcohol dehydrogenase	150,000.00	5.18	15.66	15.66	1.00
Albumin	66,000.00	4.82	17.1	15.66	1.09
Carbonic anhydrase	29,000.00	4.46	20.89	15.66	1.33
Cytochrome C	12,400.00	4.09	26.44	15.66	1.69

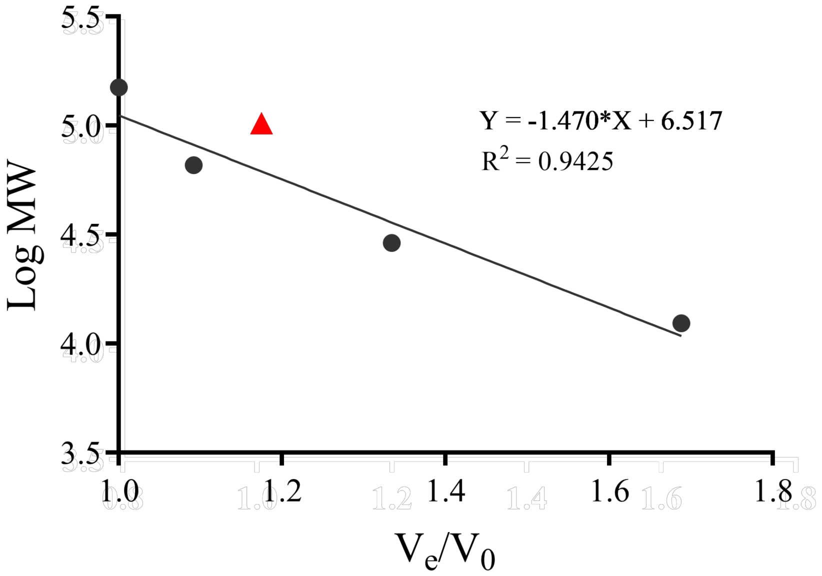

Figure S1. **A standard curve of protein markers for determination of Thal native molecular weight.** Data for the protein markers are indicated by *black circles*, while Thal is indicated by the *red triangle*.

Tryptophan chlorination activity of tryptophan halogenases.

The transient kinetics of PyrH and PrnA were investigated and compared to that of Thal. Multiple turnover reactions were performed to obtain halogenated tryptophan. The chlorination activities of PyrH, PrnA, and Thal were measured after incubation of 20 μM FAD, 20 μM NAD^+, 25 mM NaCl, 200 μM D,L-tryptophan, 20 mM HCOONa, 0.2 μM flavin reductase (C_1), 15 nM formate dehydrogenase from *Pseudomonas* sp. (psFDH) (Enzmart Co., Ltd.), 20 μM halogenases and 20 mM potassium dihydrogen phosphate buffer pH 7.2 in a total volume of 600 μL at room temperature (25-30 °C) for 2 hours on an incubator rocker set at 30 rpm. The reaction was stopped by mixing with 1 M HCl (1:1 ratio). After removal of precipitated protein by filtration, the assay mixtures were analyzed by HPLC-DAD-MS (ESI). HPLC was performed on a Zorbax Eclipse Plus C18 column (5 μm, 4.6 x 250 mm) with methanol/H_2O/formic acid (40:60:0.1) as the eluent with a flow rate of 0.9 mL/min. All HPLC grade solvents were purchased from Burdick & Jackson Scientific Co., Ltd.

To identify product from the Thal reaction, a multiple turnover reaction (38 x 1 mL) containing 10 mM potassium dihydrogen phosphate buffer pH 7.2, 20 μM FAD, 20 μM NAD^+, 12 mM NaCl, 0.8 mM D,L-tryptophan, 0.2 μM C_1, 15 nM psFDH, 22 mM HCOONa and 20 μM purified Thal was incubated at room temperature (25-30 °C) for 8 hours with rocking at 30 rpm. The reaction mixture was stopped, and protein was removed by 10 kDa cut-off filtration (Amicon® Stirred Cells). The filtrate was evaporated in a rotary vacuum. The halogenated product was purified by preparative HPLC using a Sunfire Prep C18 column (5 μm, 19 x 150 mm) with (50:50) of methanol:H_2O containing 0.1% (v/v) formic acid as the eluent at a flow rate of 3 mL/min and monitored for absorbance at 280 nm. Fractions containing product were pooled and evaporated in a rotary vacuum. The resulting aqueous solution was freeze-dried. The product was dissolved in DMSO-$_{d6}$ and analyzed by ^{1}H-NMR and ^{13}C-NMR spectroscopy in a Bruker NMR spectrometer Ascend 600 system.

Kinetics of tryptophan conversion by the three enzymes catalyzing tryptophan halogenation at different positions under multiple turnover conditions were compared. Results in Fig. S2A clearly indicate that Thal has an overall rate of tryptophan consumption similar to PyrH which is faster than PrnA. NMR spectrum of tryptophan substrate showed an H-6 peak (δ_H 7.10 ppm, *t*, J = 6.0 Hz), while the purified product from the Thal reaction showed a singlet peak for the H-7 (δ_H 7.40 ppm, *s*), implying that the product of the Thal reaction was substituted at the sixth position (Fig. S2C and Fig. S4). HPLC chromatograms (Fig. S2B) and NMR spectra (Fig. S2C and Fig. S4) of the product were consistent with those in the literature (2), clearly indicating that Thal specifically performs halogenation at the sixth-position of tryptophan. HPLC chromatograms of PrnA and PyrH reactions are shown in Fig. S3.

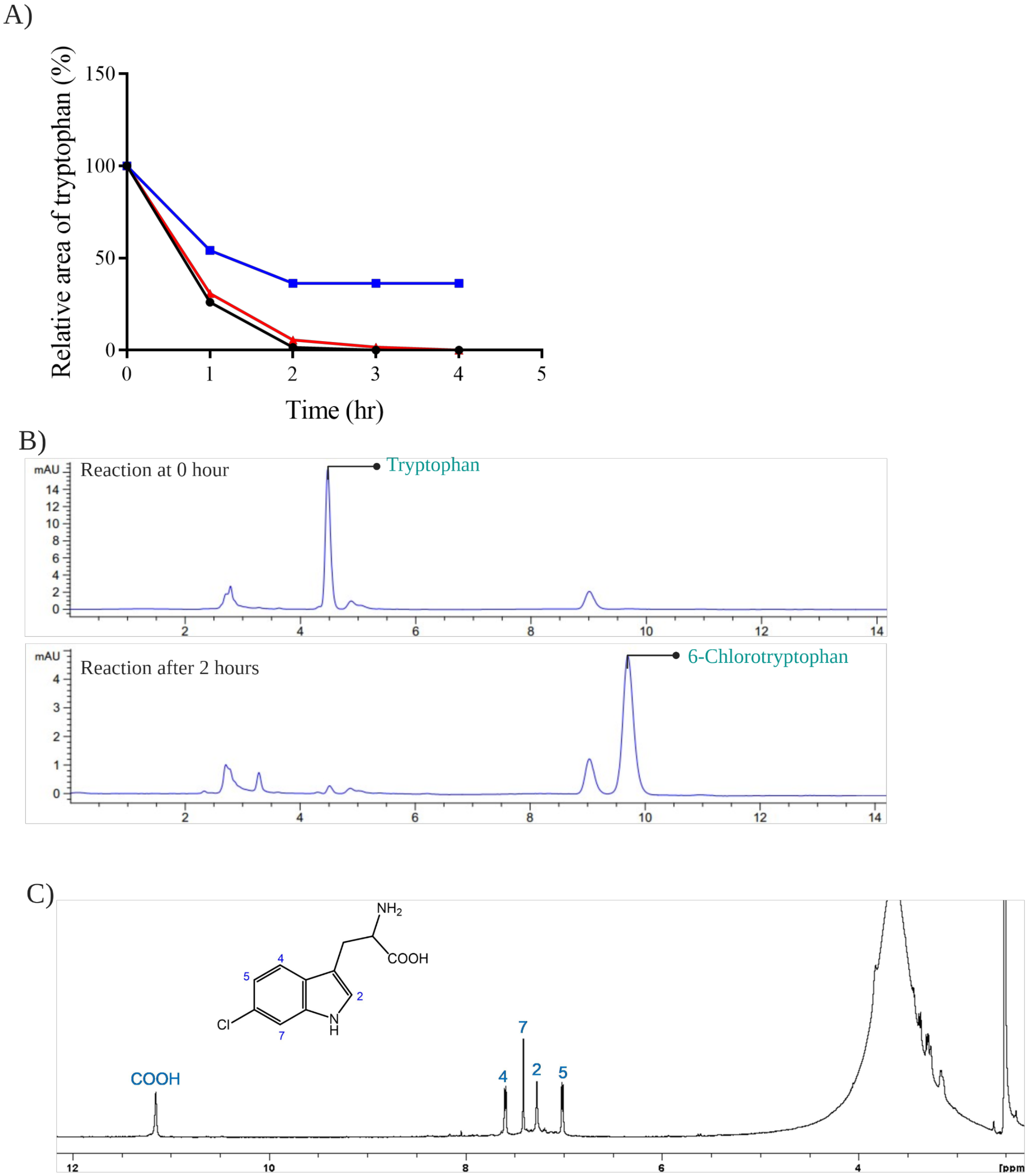

Figure S2. **Tryptophan chlorination activity of tryptophan halogenases.** Substrate decrease of Thal (*black circle*), PrnA (*blue square*) and PyrH (*red triangle*) (A). Identification of tryptophan chlorination by Thal was analyzed by high performance liquid chromatography-mass spectrometry (HPLC-DAD-MS (ESI)) (B). HPLC was performed using a Zorbax Eclipse Plus C18 column (5 µm, 4.6 x 250 mm) with methanol/H_2O/formic acid. Tryptophan (R_t = 4.4 minutes) and 6-chlorotryptophan (R_t = 9.7 minutes) were monitored at 280 nm. The peak at ~9 minutes (0 and 2 hours of reaction) is suspected to be an impurity because its maximum absorption is at 250 nm, which is not characteristic of any of the chemicals in the reaction. ^{1}H-NMR (600 MHz, Bruker) of 6-chlorotryptophan in DMSO-d_6 purified from the reaction of Thal (C).

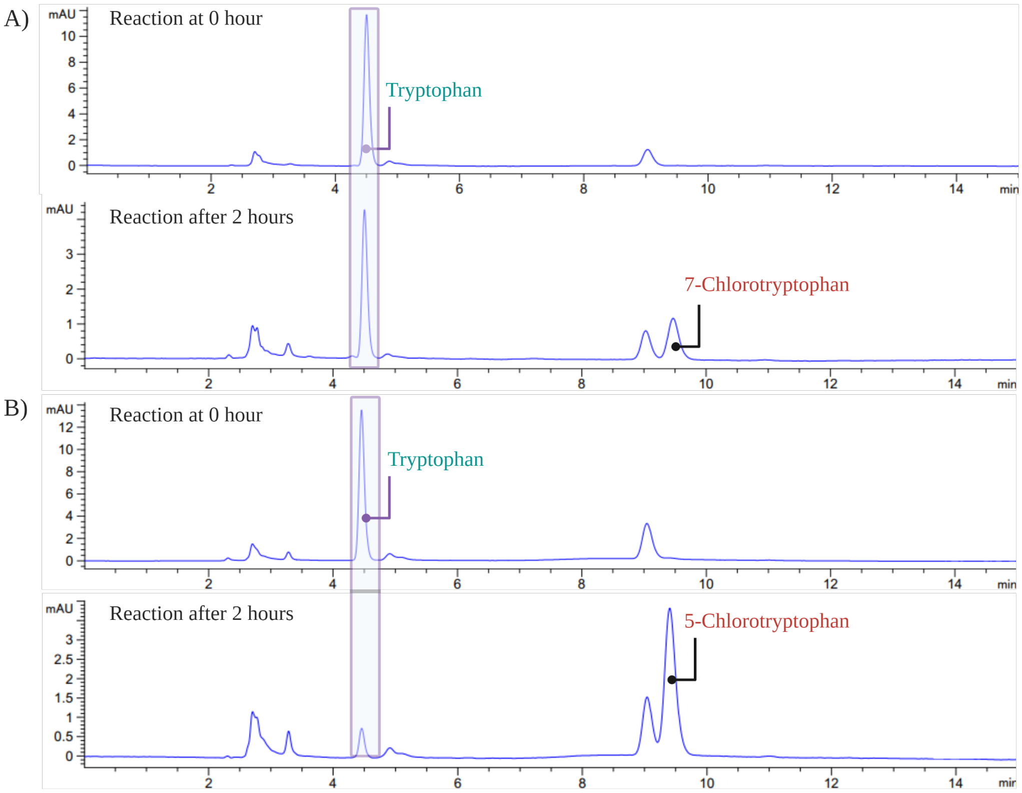

Figure S3. **Identification of tryptophan chlorination by tryptophan halogenases analyzed by HPLC-DAD-MS (ESI).** Products from the halogenase reactions were separated using a Zorbax Eclipse Plus C18 column (5 µm, 4.6 x 250 mm) with methanol/H_2O/formic acid as a mobile phase. Tryptophan chlorination by PrnA (A) and PyrH (B). Tryptophan (R_t = 4.4 minutes), 7- and 5-chlorotryptophan (R_t = 9.7 minutes) were monitored at 280 nm.

Approximately 5 mg of chlorinated tryptophan product was obtained as a yellowish solid (63% isolated yield). Fig. S4 shows ^{1}H-NMR (600 MHz, DMSO-$_{d6}$) spectra of 6-chlorotryptophan δ 7.57 (1H, *d*, J = 6.0 Hz), 7.40 (1H, *s*), 7.24 (1H, *s*), and 7.00 (1H, *d*, J = 6.0 Hz) ppm. ^{13}C-NMR (150 MHz, DMSO-$_{d6}$): δ 137.1, 126.5, 126.3, 126.2, 120.3, 119.2, 111.4, 72.9, 63.6, 54.0 and 26.8 ppm.

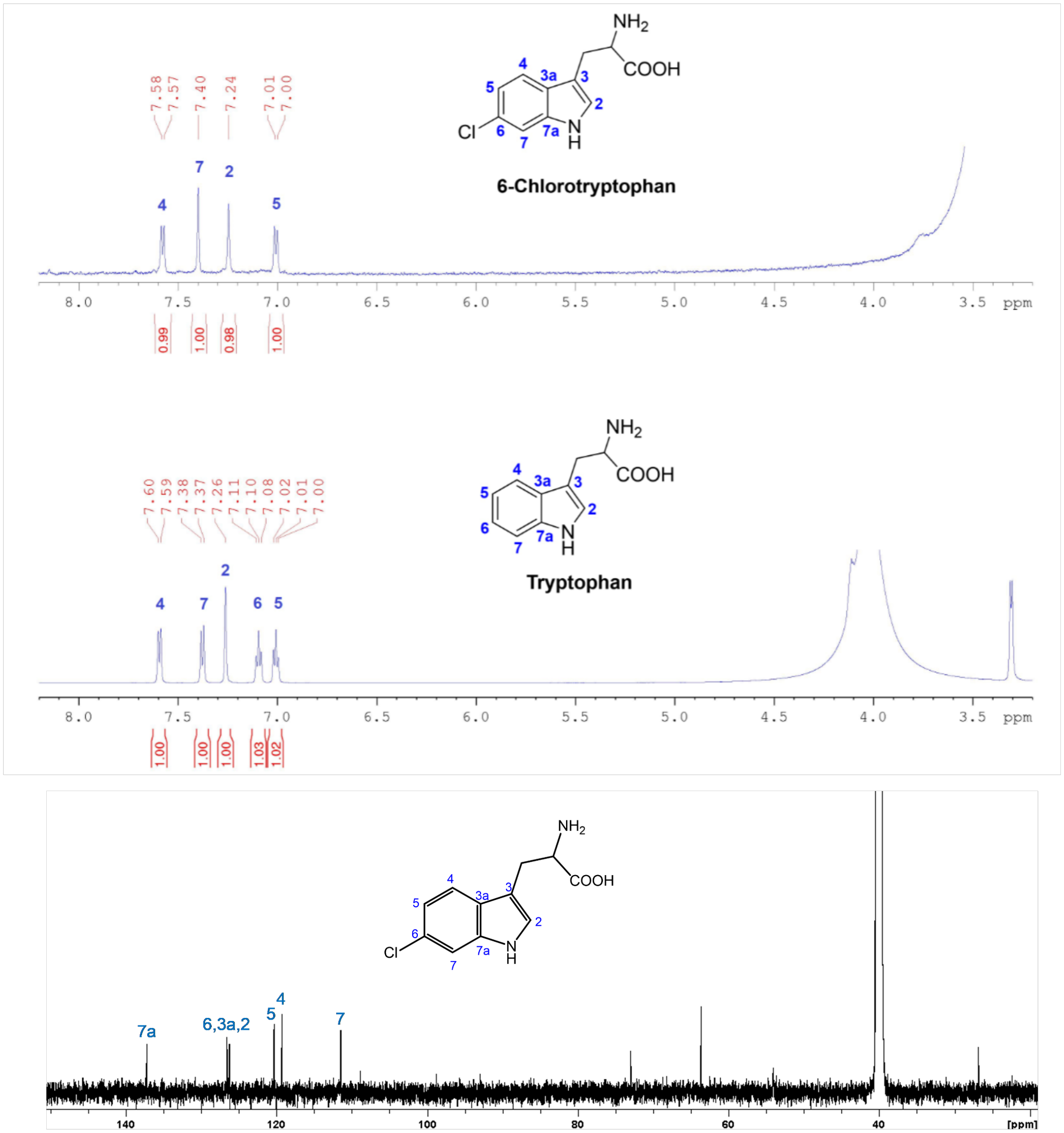

Figure S4. **NMR spectra of tryptophan and 6-chlorotryptophan.** ^{1}H-NMR (600 MHz, DMSO-$_{d6}$) spectra of 6-chlorotryptophan purified from the Thal reaction (top) and the tryptophan standard (middle). ^{13}C-NMR (150 MHz, DMSO-$_{d6}$) of 6-chlorotryptophan purified from the Thal reaction (bottom).

Transient kinetics experiments investigated by stopped-flow spectrometry.

Formation of C4aOOH-FAD can be monitored by mixing $FADH^-$ with an aerobic solution of Thal.

As Thal belongs to the class of flavin-dependent monooxygenases which uses $FADH^-$ as a substrate (3), we first assessed its ability to bind $FADH^-$ and its ability to form C4aOOH-FAD. Stopped-flow experiments were carried out to monitor C4aOOH-FAD formation at the wavelengths of 380 and 450 nm. Results in Fig. S5 indicate that a clear formation of C4aOOH-FAD could only be observed when an anaerobic solution of $FADH^-$ was mixed with an air-saturated solution of Thal (Fig. S5A), while the mixing of a preformed Thal:$FADH^-$ complex with aerobic buffer did not result in any formation of C4aOOH-FAD (Fig. S5B). The reaction showed an increase in absorbance at 380 nm during the first phase (0.02-0.13 s) and a small decrease in absorbance at 380 nm in the second phase (0.13-100 s) (Fig. S5A). For the absorbance change at 450 nm, only the second phase (0.13-100 s) showed a large absorbance increase. These data indicate that Thal quickly binds $FADH^-$ upon mixing in the stopped-flow and the Thal:$FADH^-$ complex reacts with oxygen to form C4aOOH-FAD.

When the same experiments were carried out using detection wavelengths from 300-600 nm, similar kinetics and absorbance changes were observed. A plot of the absorbance at 0.1 s at each wavelength represents the absorption spectrum of C4aOOH-FAD formed in the Thal reaction (inset of Fig. S5A). The absorption spectrum of C4aOOH-FAD detected in the Thal reaction is similar to the absorption spectra of C4aOOH-FAD found in the reaction of RebH (4) and for the other two-component flavin-dependent monooxygenases such as bacterial luciferases (5), dehalogenase (HadA) (6), *p*-hydroxyphenylacetate 3-hydroxylase (HPAH) (7), styrene monooxygenase (StyA) (8) and etc. Later, H_2O_2 is eliminated from C4aOOH-FAD to form oxidized FAD in the final step (main text, Fig.3).

Characterization of spectra of flavin intermediates.

A solution of 30 μM $FADH^-$ was mixed with an anaerobic solution of 20 mM potassium dihydrogen phosphate buffer pH 7.2 to obtain a spectrum of $FADH^-$. A solution of 30 μM $FADH^-$ was mixed with an air-saturated solution of 20 mM potassium dihydrogen phosphate buffer pH 7.2 to obtain a spectrum of FAD. To obtain a spectrum of the C4aOOH-FAD intermediate, an air-saturated solution of 60 μM of Thal was mixed with a solution of 30 μM $FADH^-$. Reactions were monitored using the diode array mode of a double mixing stopped-flow spectrophotometer. The spectra of flavin intermediates were analyzed using the ReactLab KINETICS program.

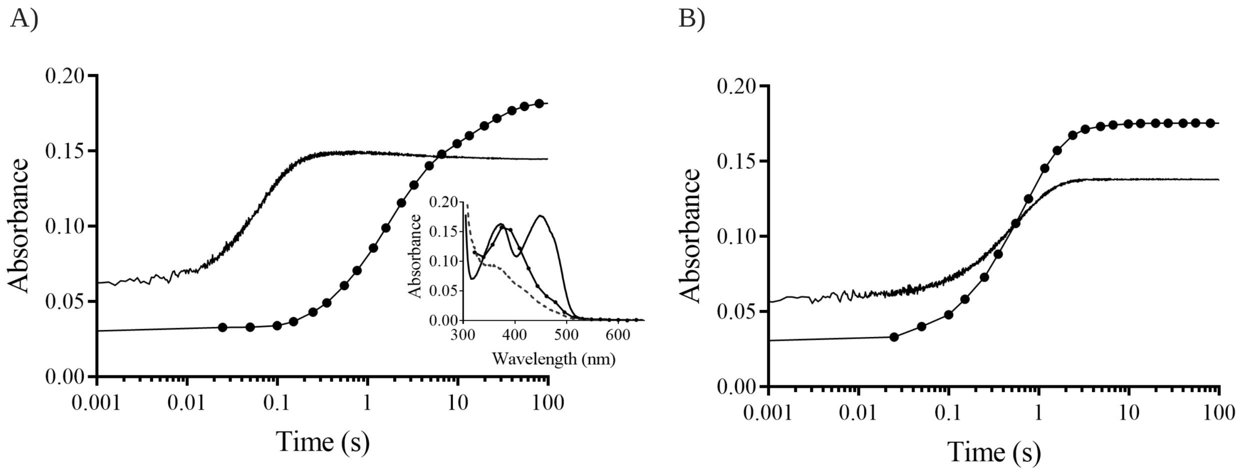

Figure S5. **Reaction of Thal:FADH⁻ with oxygen.** Kinetic traces were monitored at 380 nm (*solid lines*) and 450 nm (*solid lines with circles*). An air-saturated solution of Thal (30 μM) was freshly mixed with a solution of FADH⁻ (15 μM) (A). Spectra of flavin intermediates: FADH⁻ (*dashed line*), C4aOOH-FAD (*solid line with circles*), FAD (*solid line*) (inset of A). An anaerobic solution of pre-mixed Thal (30 μM) and FADH⁻ (15 μM) was mixed with a solution of oxygen (B). The concentrations indicated are concentrations after mixing.

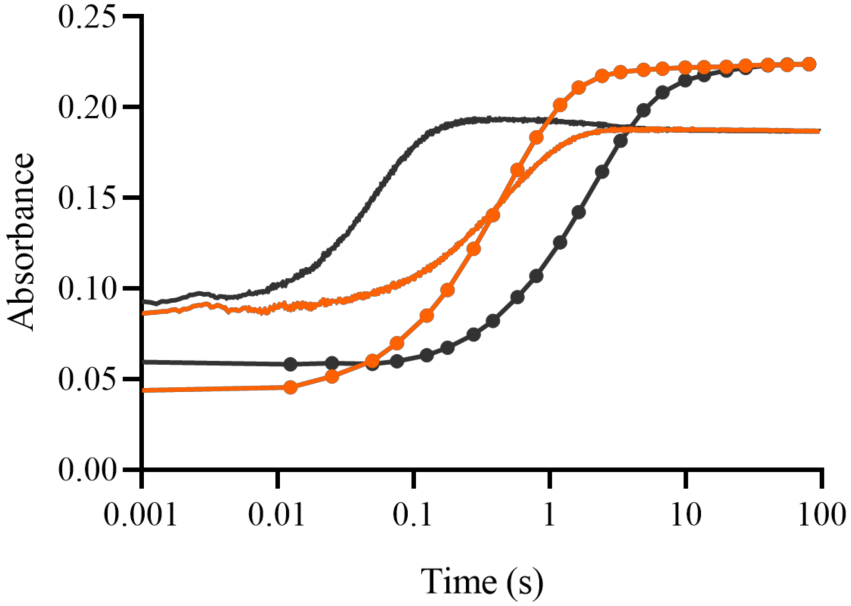

Figure S6. **Reaction of Thal:FADH⁻ with oxygen under light-minimization conditions.** Reactions were performed as in Fig. S5, but with sample preparation carried out strictly under darkness in order to identify whether the inactivation process was due to light inactivation. Kinetic traces were monitored at 380 nm (*solid lines*) and 450 nm (*solid lines with circles*). An air-saturated solution of Thal (30 μM) was freshly mixed with a solution of FADH⁻ (15 μM) (*black*). An anaerobic solution of pre-mixed Thal and FADH⁻ was mixed with a solution of oxygen (*orange*). The concentrations indicated are concentrations after mixing.

Identification of the H_2O_2 elimination path.

Air-saturated solutions containing Thal with and without halide ions were shot against an $FADH^-$ solution in 20 mM potassium dihydrogen phosphate buffer pH 7.2. Final concentrations were 10 mM salt (NaBr or NaCl), 128 µM O_2, 15 µM $FADH^-$ and 30 µM Thal. Flavin intermediates were monitored using absorption mode at 380 nm and 450 nm.

Investigation of Thal:$FADH^{-*}$ inactive complex formation.

Previous studies reported that premixing of tryptophan 7-halogenase (RebH) and $FADH^-$ prevents C4aOOH-FAD formation (4). To examine whether Thal can form an inactive complex with $FADH^-$, the reaction of pre-incubated Thal and $FADH^-$ was compared to the reaction of freshly mixed Thal and $FADH^-$ in 20 mM potassium dihydrogen phosphate buffer pH 7.2. A pre-incubated solution of Thal and $FADH^-$ in one syringe was mixed with an air-saturated buffer from a second syringe. For the freshly mixed reaction, air-saturated buffer containing Thal was mixed with $FADH^-$. Final concentrations were 128 µM O_2, 15 µM $FADH^-$ and 30 µM Thal. Flavin intermediates were monitored using absorption mode at 380 nm and 450 nm.

Determination of the formation rate of the inactive complex.

Anaerobic solution of Thal was prepared by exposing the solution to nitrogen gas in a glove box for 1 hour. The enzyme solution was then transferred to a syringe. The syringe was a closed system to prevent oxygen from entering the solution. For the first mixing, the $FADH^-$ solution was mixed with an anaerobic solution of Thal in 20 mM potassium dihydrogen phosphate buffer pH 7.2. Age time (incubation time) of the first mix was varied from 0.01-30 second. Aerobic buffer was then added at the second step. Final concentrations were 128 µM O_2, 15 µM $FADH^-$ and 30 µM Thal. Flavin intermediates were monitored using absorption mode at 380 nm and 450 nm.

Investigation of halide utilization of tryptophan halogenases.

Air-saturated solution containing Thal with various salts (NaF, NaI, KBr, or NaCl) were shot against the $FADH^-$ solution. Final concentrations were 5 mM halide salt, 128 µM O_2, 15 µM $FADH^-$ and 30 µM tryptophan halogenase (Thal, PrnA or PyrH). The C4aOH-FAD intermediate was observed using the fluorescence detection mode. Reactions were excited at 380 nm and 450 nm and emission light of wavelengths greater than 495 nm was collected.

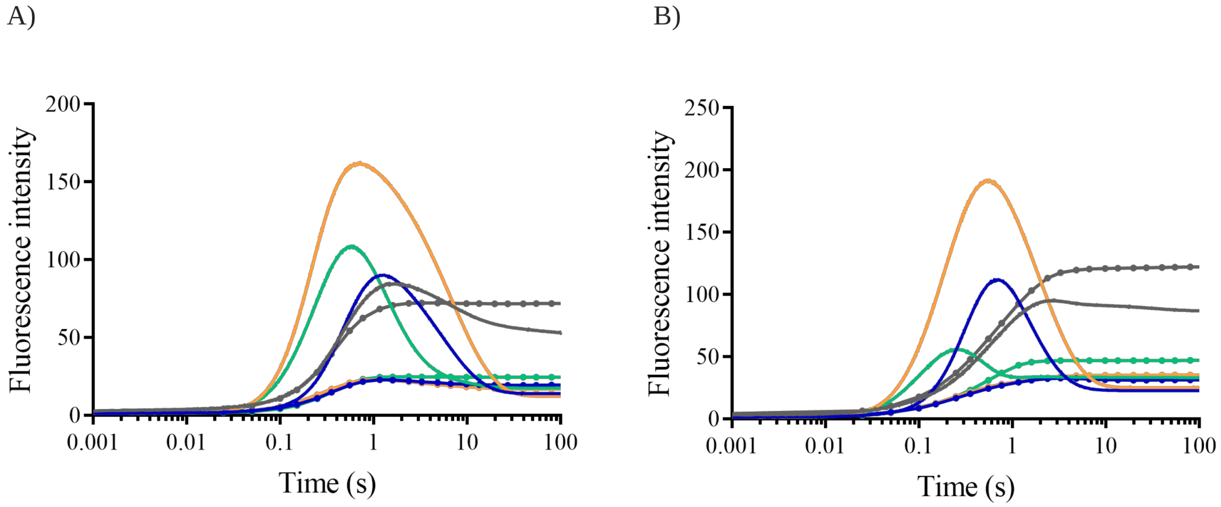

Figure S7. **Kinetic traces of PrnA (A) and PyrH (B) reactions with various halide ions.** Formation of C4aOH-FAD and oxidized FAD were monitored using excitation wavelengths of 380 nm (*solid lines*) and 450 nm (*solid lines with circles*), respectively with emission wavelengths (E_m) > 495 nm in the presence of various halide ions (Cl^- (*blue*), Br^- (*orange*) and I^- (*green*)). *Gray lines* are reaction without addition of halide ion. Note that different reactions used different PMT voltages, causing the magnitude of the fluorescence signal to be arbitrary.

Kinetic mechanisms of hypohalous acid formation.

An air-saturated solution containing Thal with various concentrations of halide salts was shot against $FADH^-$ in 20 mM potassium dihydrogen phosphate buffer pH 7.2. Final concentrations were 0.05-20 mM salt (NaI, KBr, or NaCl), 128 µM O_2, 15 µM $FADH^-$ and 30 µM Thal. An C4aOH-FAD intermediate was observed using the fluorescence detection mode. Excitation wavelengths were 380 nm and 450 nm and emission light of wavelengths greater than 530 nm was collected.

Product analysis of tryptophan halogenation by Thal.

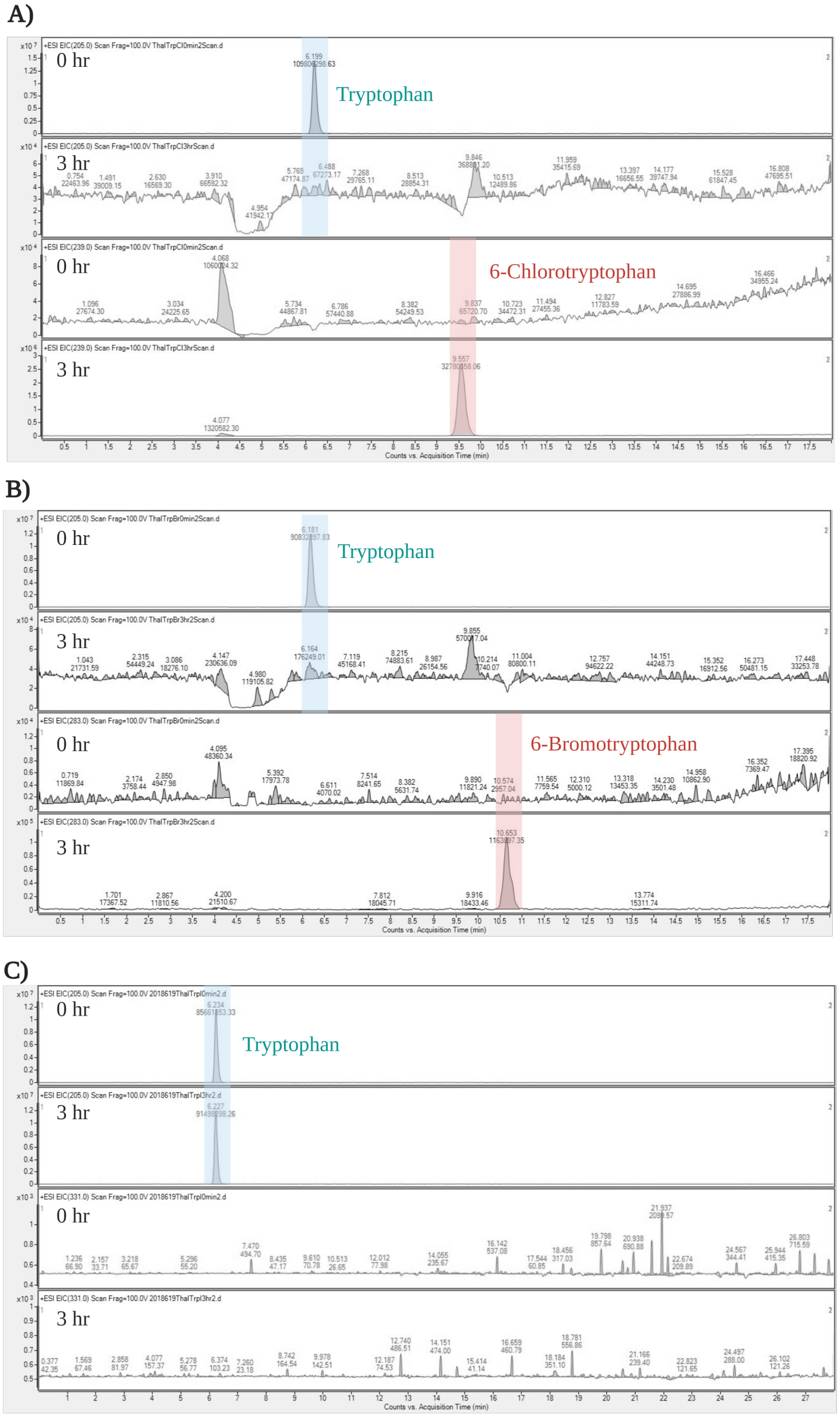

Figure S8. **Product analysis of tryptophan halogenation by Thal.** HPLC chromatograms of tryptophan chlorination (A), bromination (B) and iodination (C) monitored by HPLC-MS/MS. HPLC was performed using a Zorbax Eclipse Plus C18 column (5 µm, 4.6 x 250 mm) with water and methanol containing formic acid (0.1% v/v) as the mobile phase. Positive mode was used on the mass spectrometer for product analysis.

QM/MM calculations to explain the reactivity of C4aOOH-FAD towards different halide ions.

Potential energy surface (PES) calculations.

Although flavin-dependent tryptophan halogenases have been investigated in various experimental studies (4,9-14), to the best of our knowledge, there are only a few computational studies (15,16). To obtain an in-depth understanding of enzyme mechanism in terms of both structural and electronic functions, a combination of computational and experimental data are needed. Therefore, we combined investigation of QM/MM calculations and experimental studies in this work. The Thal structure reported in the main text (PDB code 7CU1) was used for QM/MM calculations. This structure is a crystal structure of the Thal complex with flavin adenine dinucleotide (FAD). The structure had FAD and AMP bound. The system setup was performed using the same approach as our previous modeling (17). The AMP was removed from the starting structure and replaced by water molecules in the solvation procedure. The presence of Cl^- is needed in the active site in order to calculate the formation of HOCl by the enzyme. The position of the Cl^- was identified from the Cl^- position in the PDB structure of RebH in complex with L-tryptophan and FAD (PDB code 2OA1) (18). The halide ions were added based on previous experimentally determined structural information from the structure of RebH (a FAD-dependent halogenase bound to L-tryptophan and FAD). It is also important to note that the relative reactivity of the different halide ions (which is the most important property tested here) will not be affected by small changes in position. The structures of the transition state structures were optimized at a high level, which means that the interatomic distances are likely to be predicted accurately. The calculated barriers are consistent with experimental findings. The Cl^- was docked in to the 7CU1 structure by considering relative positions of Cl^- in the 2OA1 structure. The position was found to be maintained in molecular dynamics simulations, indicating that this model represents a stable reactant complex. The C4aOOH-FAD structure was generated from the FAD structure with OOH attached at the C4a atom of the FAD. The starting structure was equilibrated with QM/MM molecular dynamics (MD) to release strain in the starting structure. In order to model the reaction mechanism of HOCl formation, the reaction mechanism was designed and a potential reaction mechanism was hypothesized in Fig. S9. The system was truncated to a 25 Å sphere with the center at the carbon atom C4a of FAD molecule (see Fig. S9). The positions of hydrogen atoms were located in the enzyme using the CHARMM procedure HBUILD (19). Protonation states of amino acid residues were assigned using a method that is simple, fast and widely used in the setup of proteins; propka (20). Hydrogen atoms of the amino acid residues were added by considering results from the propka analysis (20). The atom types in the topology files were assigned based on the CHARMM27 parameter set (21). The system was divided into two parts, which are QM and MM parts for QM/MM simulations. The QM part consisted of Cl^- and C4aOOH-FAD. C4aOOH-FAD was partitioned across the bond between atoms C1' and C2' by adding a link atom (22) between these atoms. Therefore, the QM region consisted of 37 atoms and had a net charge of 0e. The model was divided into two zones, which are the reaction zone and the buffer zone. The reaction zone is an area with a radius of 21 Å, centered on the C4a carbon atom of the C4aOOH-FAD molecule and the buffer zone consisted of the rest of the protein system. Atoms within the reaction zone were unconstrained, while atoms within the buffer region were restrained harmonically to their initial positions in the crystal structure with force constants, which depend on the distance from the center of the system and the type of atoms. The system was minimized using 1000 steps of Adopted Basis Newton-Raphson (ABNR) minimization with the AM1/CHARMM27 method. Next, AM1/CHARMM27 molecular dynamics, using the leapfrog Langevin dynamics with a time step of 0.001 ps was performed at 300 K. The system was equilibrated with QM/MM MD for 20 ps. Coordinate driving method was used to model the reaction mechanism with two reaction coordinates. The reaction coordinate for the first step, C4aOOH-FAD-Cl formation ($R_{Cl\text{-}O2}$) is defined as $R_{Cl\text{-}O2}$ = d(Cl-O2) where d is the distance between the two atoms. The second step is the release of HOCl ($R_{O1\text{-}O2}$). For the second step, the reaction coordinate is defined by $R_{O1\text{-}O2}$ = d(O1-O2). The calculation

begins with reactant at $R_{Cl\text{-}O2}$ = 2.8 Å and continuously decreases until reaching 1.9 Å. Next, the $R_{O1\text{-}O2}$ was calculated from 1.6 Å to 2.7 Å.

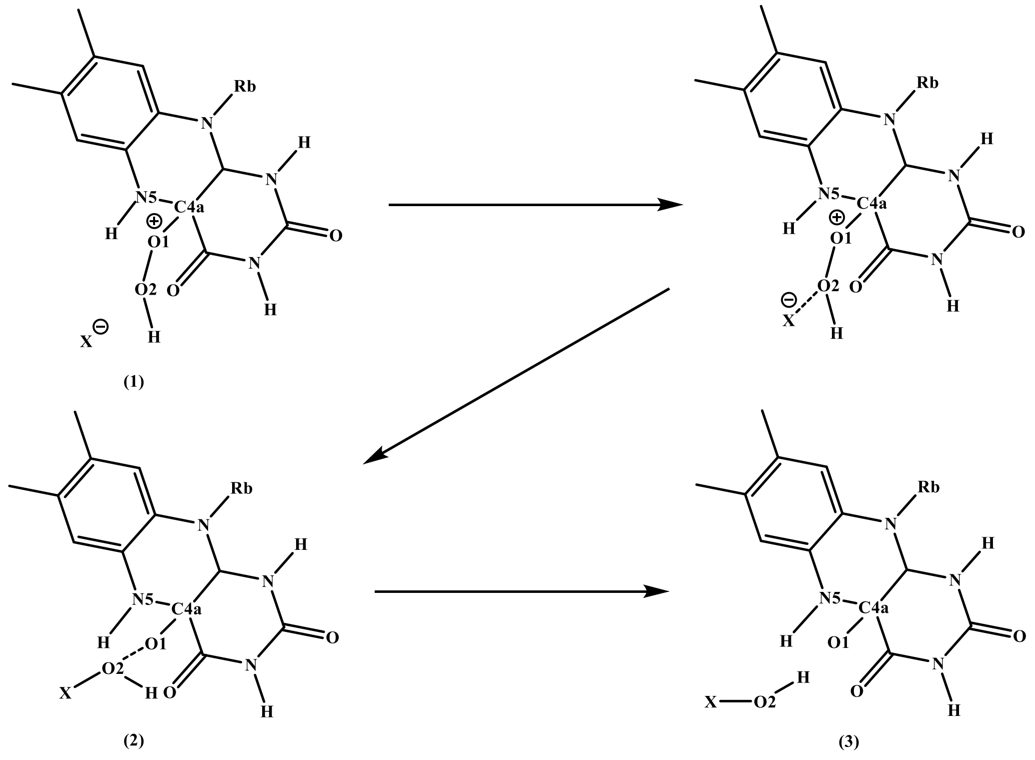

Figure S9. **Reaction mechanism for the HOX (X = I, Br or Cl) formations catalyzed by flavin-dependent halogenases (4,13), (1) reactant, (2) transition state (TS), and (3) products.**

MP2/6-31G(d)/CHARMM27 calculations (23-26), were performed with the QoMMMa program (27). The 20 ps equilibrated (AM1/CHARMM27 QM/MM MD) structure was used as the starting structure for the MP2/6-31G(d)/CHARMM27 calculations. This program is a code linking results from Gaussian09 program (28) for quantum mechanics (QM) and Tinker program (29) for molecular mechanics (MM) calculations. The Cl^- anion and C4aOOH-FAD molecule were treated as the QM part. The rest of the protein and water molecules were treated as the MM part. The MM part was assigned and treated based on the CHARMM27 parameter set (21). A full optimization at the MP2/6-31G(d)/CHARMM27 level was applied at each point along the reaction coordinates of $R_{Cl\text{-}O2}$ and $R_{O1\text{-}O2}$. The reaction coordinates were restrained with a harmonic force constant for adiabatic mapping of 1,000 kcalmol^{-1}Å^{-2}. The HOBr and HOI formation reactions were modelled using the same procedures as the HOCl formation calculation. High-level QM/MM potential energy surfaces were calculated from these structures with the SCS-MP2 method, which has been shown to give accurate results, close to coupled cluster calculations, for reaction barriers and energies of other enzyme-catalysed reactions (30-33). SCS-MP2/aug-cc-pvdz/CHARMM27 (HOCl and HOBr formations) and SCS-MP2/aug-cc-pvdz,I=auc-cc-pwcvdz-pp/CHARMM27 (HOI formation) calculations (auc-cc-pwcvdz-pp with the corresponding pseudopotential was used for iodine) were carried out using the Molpro program (34) on the optimized structure from MP2/6-31G(d)/CHARMM27 (HOCl and HOBr formations) and MP2/6-31G(d),I=lanl2dz/CHARMM27 (HOI formation) calculations (lanl2dz with the corresponding pseudopotential was used for iodine). In order to compare the activities of the enzyme for HOCl, HOBr and HOI formation, the Cl^- anion was replaced by Br^- and I^- for HOBr and HOI formation calculations, respectively. Chemical structures of the reactant, transition state and products for the HOCl, HOBr and HOI formation reactions are shown in Fig. S10, S11 and S12, respectively. SCS-MP2/aug-cc-pvdz,I=auc-cc-pwcvdz-pp/CHARMM27 PESs for Thal catalyzed the HOCl, HOBr and HOI formations are shown in Fig. S13, S14 and S15, respectively.

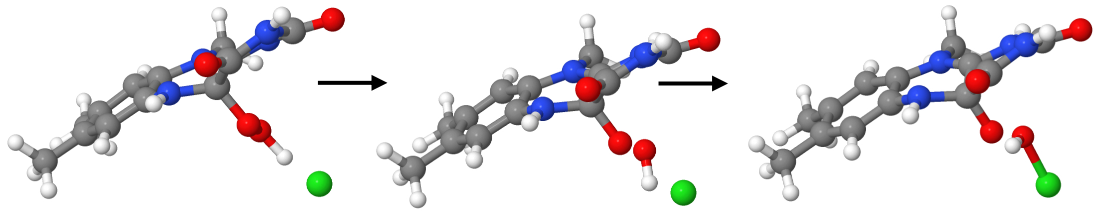

(1) Reactant (2) TS (3) Product

Figure S10. **Chemical structures of C4aOOH-FAD and Cl^- along the reaction generated from MP2/6-31G(d)/CHARMM27 calculations.**

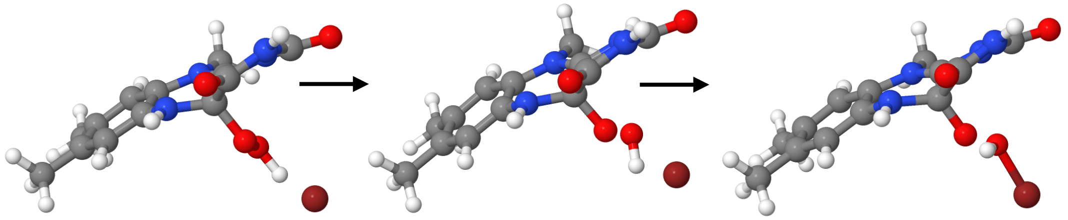

(1) Reactant (2) TS (3) Product

Figure S11. **Chemical structures of C4aOOH-FAD and Br^- along the reaction generated from MP2/6-31G(d)/CHARMM27 calculations.**

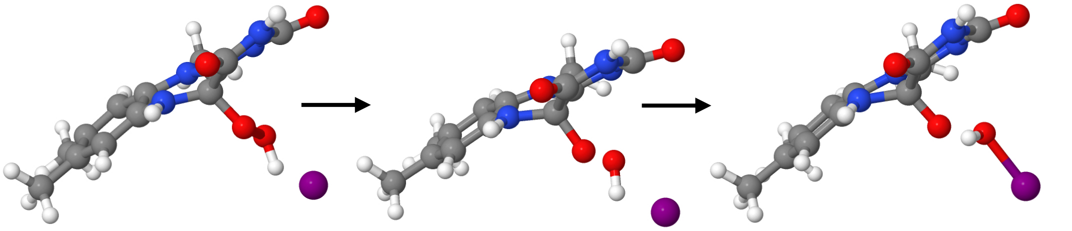

(1) Reactant (2) TS (3) Product

Figure S12. **Chemical structures of C4aOOH-FAD and I^- along the reaction generated from MP2/6-31G(d),I=lanl2dz/CHARMM27 calculations.**

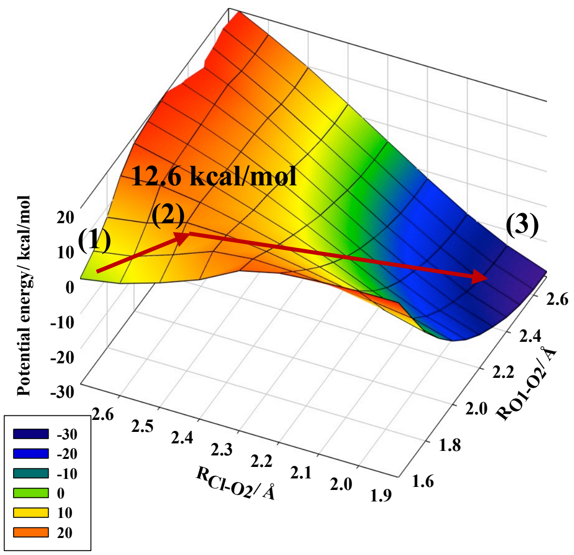

Figure S13. **SCS-MP2/aug-cc-pvdz/CHARMM27 PES for HOCl formation.** The concerted reaction composed of (i) C4aOOH-FAD-Cl formation, corresponding to Cl-O2 bond forming ($R_{Cl\text{-}O2}$) and (ii) HOCl release, corresponding to O1-O2 bond breaking ($R_{O1\text{-}O2}$), (1) reactant, (2) transition state (TS), and (3) products.

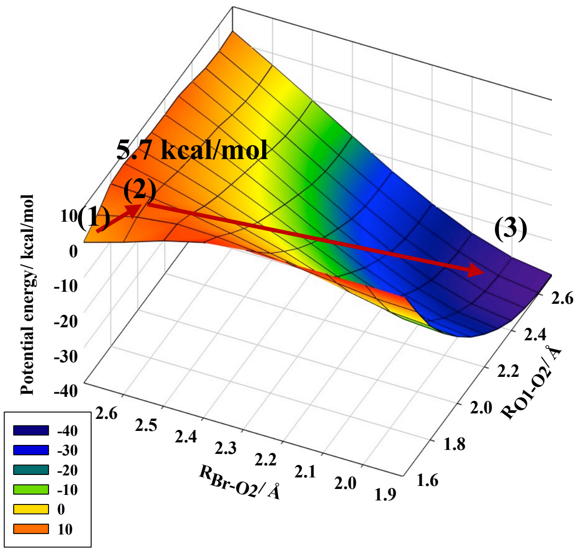

Figure S14. **SCS-MP2/aug-cc-pvdz/CHARMM27 PES for HOBr formation.** The concerted reaction composed of (i) C4aOOH-FAD-Br formation, corresponding to Br-O2 bond forming ($R_{Br\text{-}O2}$) and (ii) HOBr releasing, corresponding to O1-O2 bond breaking ($R_{O1\text{-}O2}$), (1) reactant, (2) transition state (TS), and (3) products.

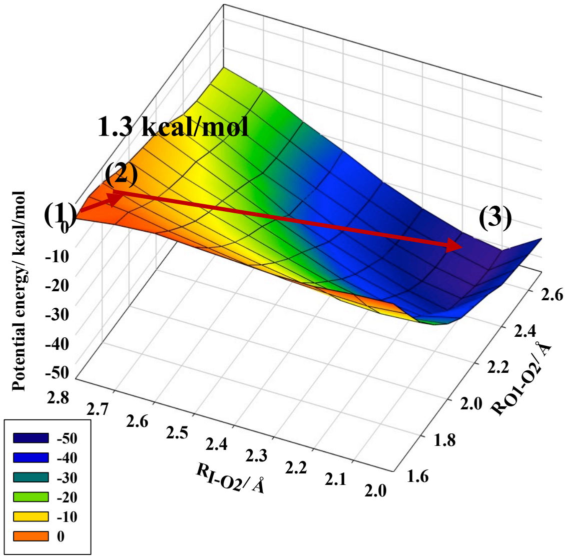

Figure S15. **SCS-MP2/aug-cc-pvdz,I=auc-cc-pwcvdz-pp/CHARMM27 PES for HOI formation.** The concerted reaction mechanism composed of (i) C4aOOH-FAD-I formation, corresponding to I-O2 bond forming (R_{I-O2}) and (ii) HOI releasing, corresponding to O1-O2 bond breaking (R_{O1-O2}), (1) reactant, (2) transition state (TS), and (3) products.

Mulliken charge analysis.

Mulliken charge analysis for HOCl formation catalyzed by Thal is shown in Fig. S16. For the first step, Cl lost some electrons as the charge changed from -0.75 to -0.32 e, while O1 received more electrons as its charge changed from -0.49 to -0.76 e. O2 lost some electrons at the initial step, and received some electrons back at the end of the first step. For the second step, Cl lost its electrons as its charge changed from -0.32 to 0.13 e while O2 received more electron as its charge changed from -0.42 to -0.74 e. O1 received some electrons as its charge changed from -0.76 to -0.84 e.

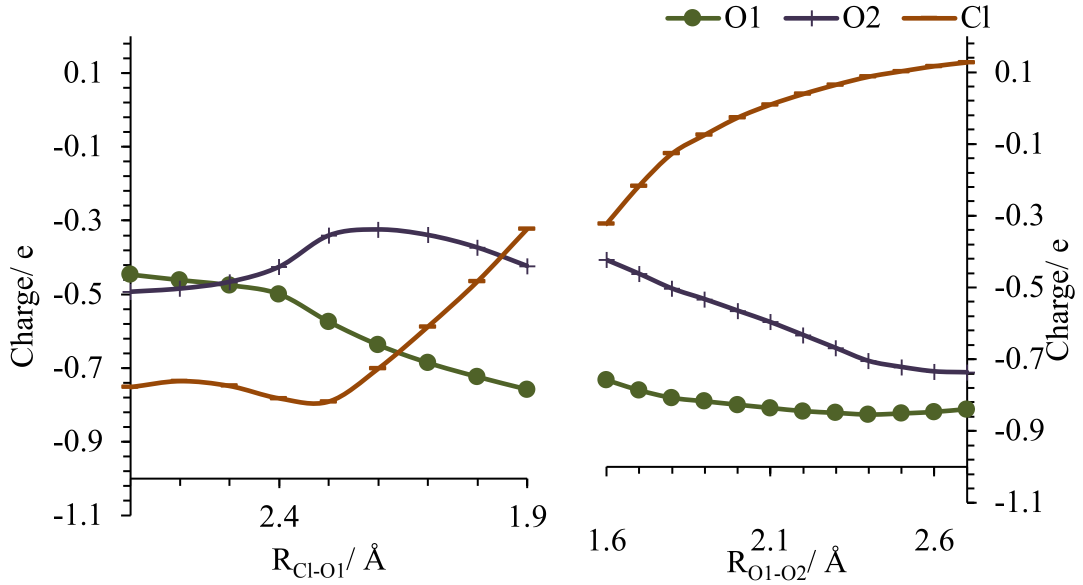

Figure S16. **Mulliken charge analysis for the HOCl formation catalyzed by Thal along R_{Cl-O2} and R_{O1-O2}, using MP2/aug-cc-pvdz/CHARMM27 calculations.**

Site-saturation mutagenesis at position Lys79^{Thal}.

Table S2.

Primers for construction of Lys79 mutant libraries.

Primers	Nucleotide sequences
Forward	5’-CGAGCTATNNKATGGCGGTTAAATTCATTAACTGGCGTAC-3’
Reward	5’-AATTTAACCGCCATMNNATAGCTCGCGTTGCATTCAC-3’

Table S3.

Amount of Thal enzyme obtained after purification.

Enzyme	Enzyme obtained per gram cells (mg/g)
Wild-type	0.56
Lys79Ser	1.64
Lys79Thr	1.76
Lys79Asn	0.26

Table S4.

p*K*a of Lys residues in Chain A and Chain B of Thal (PDB code 7CU1) calculated by propka3.0 (35).

Residue Number	p*K*a (Chain A)	p*K*a (Chain B)
6	10.80	10.43
26	10.25	10
33	10.85	10.88
79	7.48	7.59
83	9.01	8.73
130	10.78	11.02
154	10.00	10.15
155	10.11	9.83
176	10.39	11.09
188	10.44	10.34
215	10.64	10.7
238	9.77	10.34
267	10.56	10.52
286	10.60	9.8
315	11.40	11.03
374	9.41	11.51
379	10.57	10.68
383	10.53	10.33
423	9.22	10.5
426	10.48	10.41
432	10.67	10.61
434	10.43	9.97
492	9.57	9.69
507	11.15	11.16
509	10.29	10.14

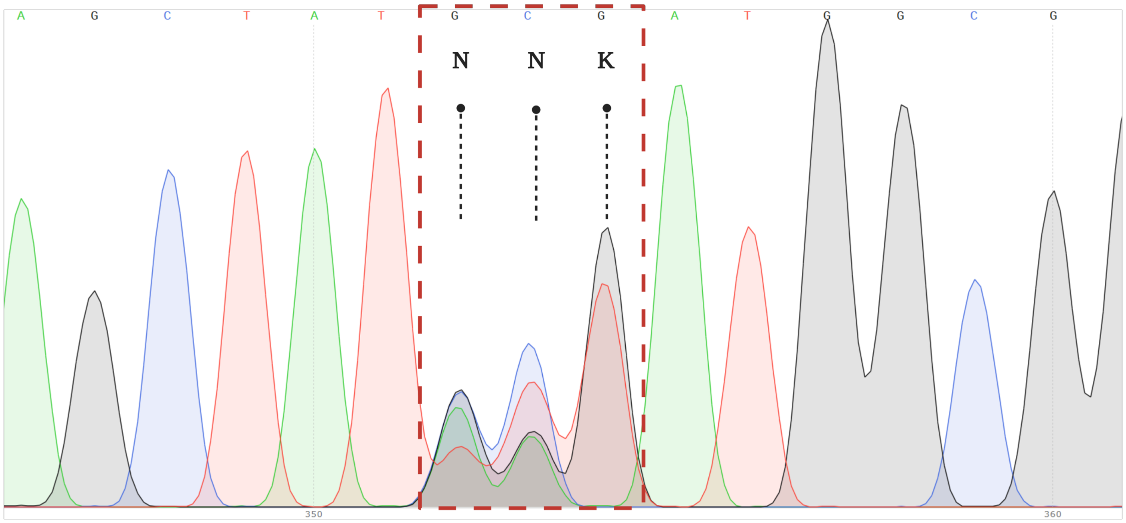

Figure S17. **DNA sequencing read by fluorescence of site-saturation mutagenesis of a Thal library showing 4-4-2 (NNK) nucleotide distribution at the Lys79 position.**

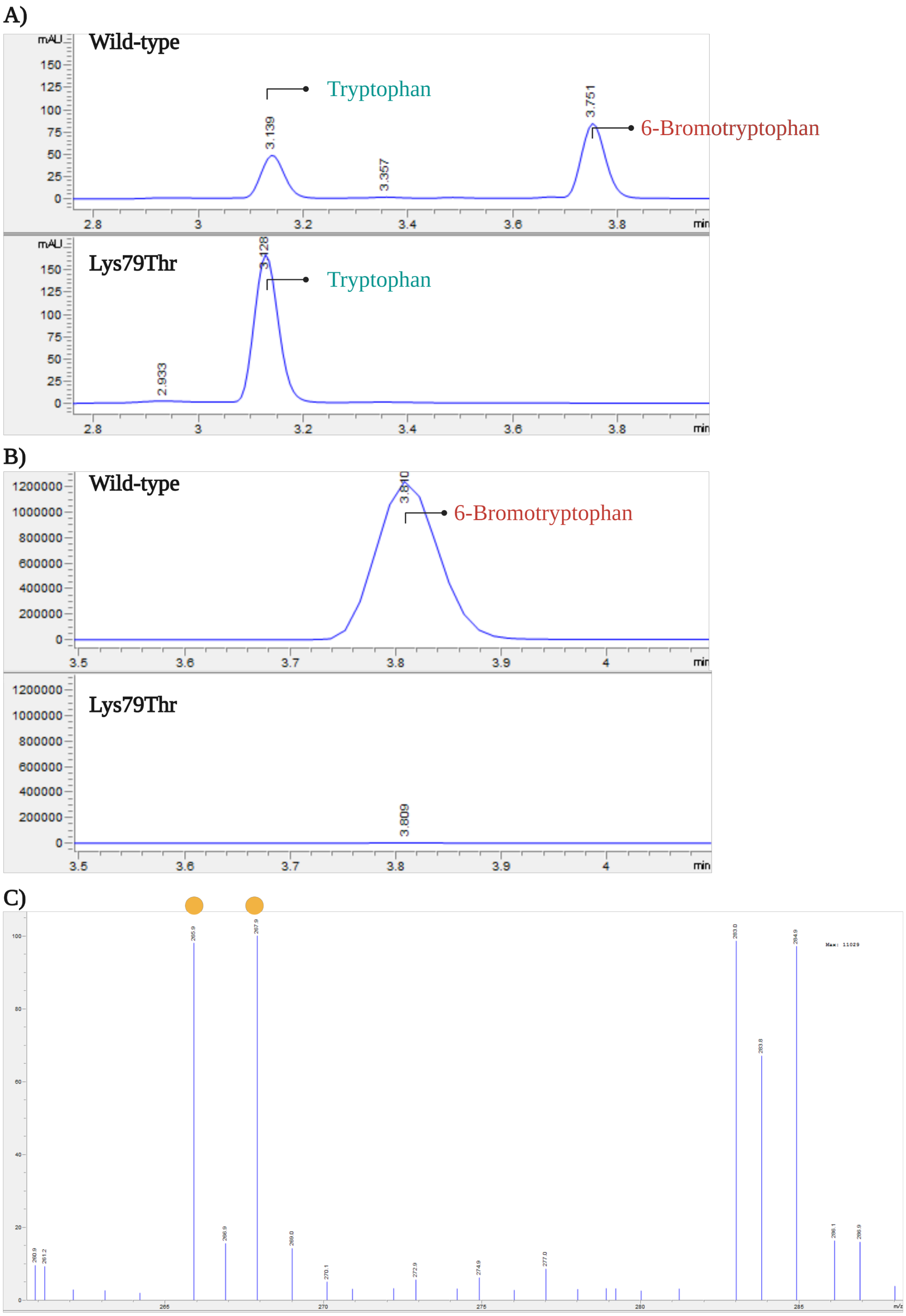

Figure S18. **Tryptophan bromination activity of Thal wild-type and Lys79Thr variants.** HPLC chromatograms of Thal reactions monitored using a DAD detector (A). HPLC chromatogram of Thal reactions monitored using a mass spectrometer (B). Mass spectrum of 6-bromotryptophan product (C).

Crystallographic ligand density in Thal structures.

A)

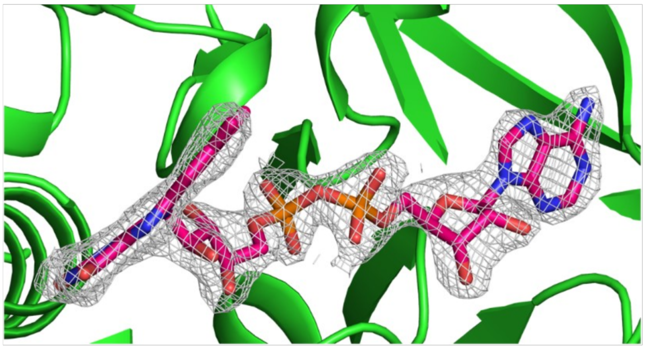

B)

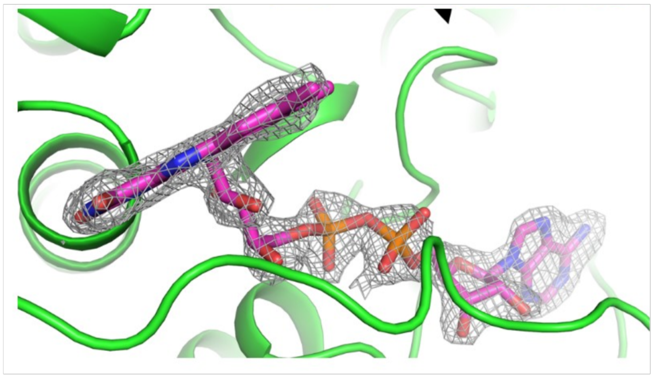

Figure S19. **Electron density of FADH$^-$ and FAD.** $2F_0 - F_c$ electron density map from crystallography represented as gray mesh is contoured at 1.5 σ FADH$^-$ (A) and at 1.2 σ for FAD (B).

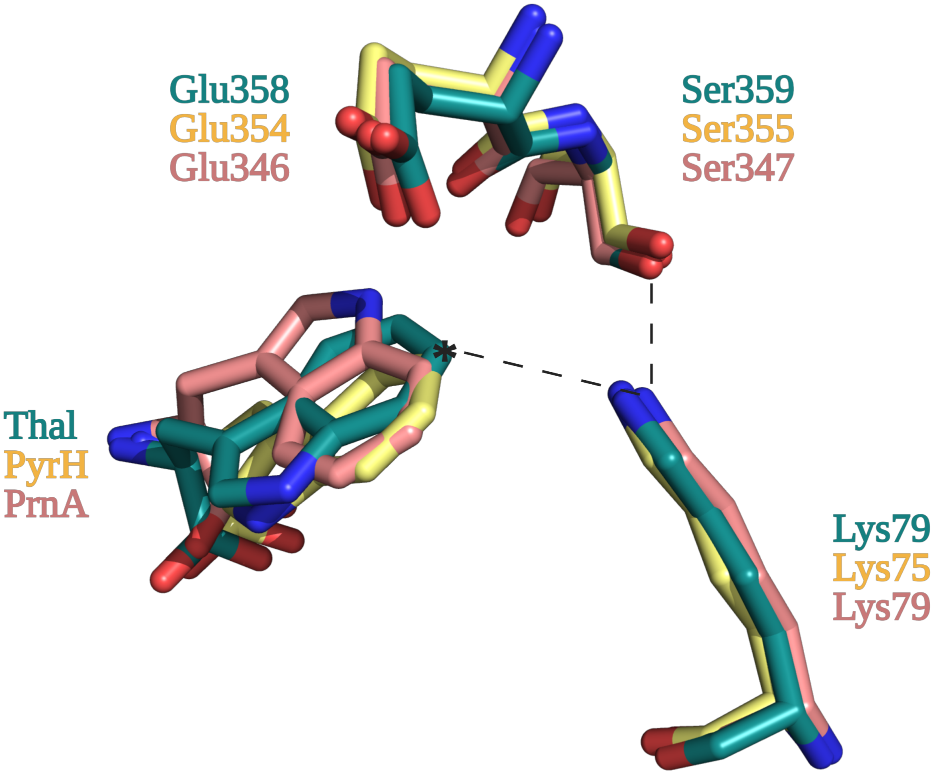

Figure S20. **Tryptophan arrangement for regioselectivity in Thal, PrnA and PyrH.** Overlay of the tryptophan substrate showing the halogenated position facing Lys, Glu and Ser of Thal (PDB code 7CU0), PrnA (PDB code 2AQJ) and PyrH (PDB code 2WEU).

Conformation changes of Thal explain formation of inactive complex.

Alignment of Thal:FADH$^-$ (PDB code 7CU2) and Thal:FAD:AMP (PDB code 7CU1) showed that Thal has a high degree of conformational heterogeneity, especially at the loop around residues 39-53 (defined as the flavin binding loop) which closely interacts with the flavin (main text, Fig. 10). Each enzyme conformation has different interactions with flavin. The conformation of the flavin binding loop (blue color, residue 39-53) observed in Chain A of Thal:FADH$^-$ (PDB code 7CU2) is referred to as the "loose conformation" because only a positively charged Arg44 on the blue loop interacts with a negatively charged phosphate group of FADH$^-$ (Fig. S21A). The conformation of the blue loop observed in Chain B of Thal:FADH$^-$ (PDB code 7CU2) is referred to as the "tight conformation" because this conformation holds FADH$^-$ tighter than the loose conformation with the blue loop forming multiple interactions with FADH$^-$ (Fig. S21B and S21C). For the tight conformation, the backbone of Glu49 forms hydrogen bonds (distance of 2.7 and 2.7 Å) with a backbone of Phe172 (Fig. S21C). The side chain of Glu49 forms a hydrogen bond (2.6 Å) with FADH$^-$ (Fig. S21C). For Chain A of Thal:FAD:AMP (PDB code 7CU1), only a carbonyl group of the back bone of Glu49 is located close to (3.0 Å) the backbone of Phe172. The side chain of Glu49 flips down to form a salt bridge (3.1 Å) with Arg292 instead (Fig. S21D). This conformation is referred to as the "open conformation" in which the conformational change of residues 45-49, especially Glu49, creates a network of water which may serve as a proton transfer pathway connecting the active site of Thal with bulk solvent (main text, Fig. 11).

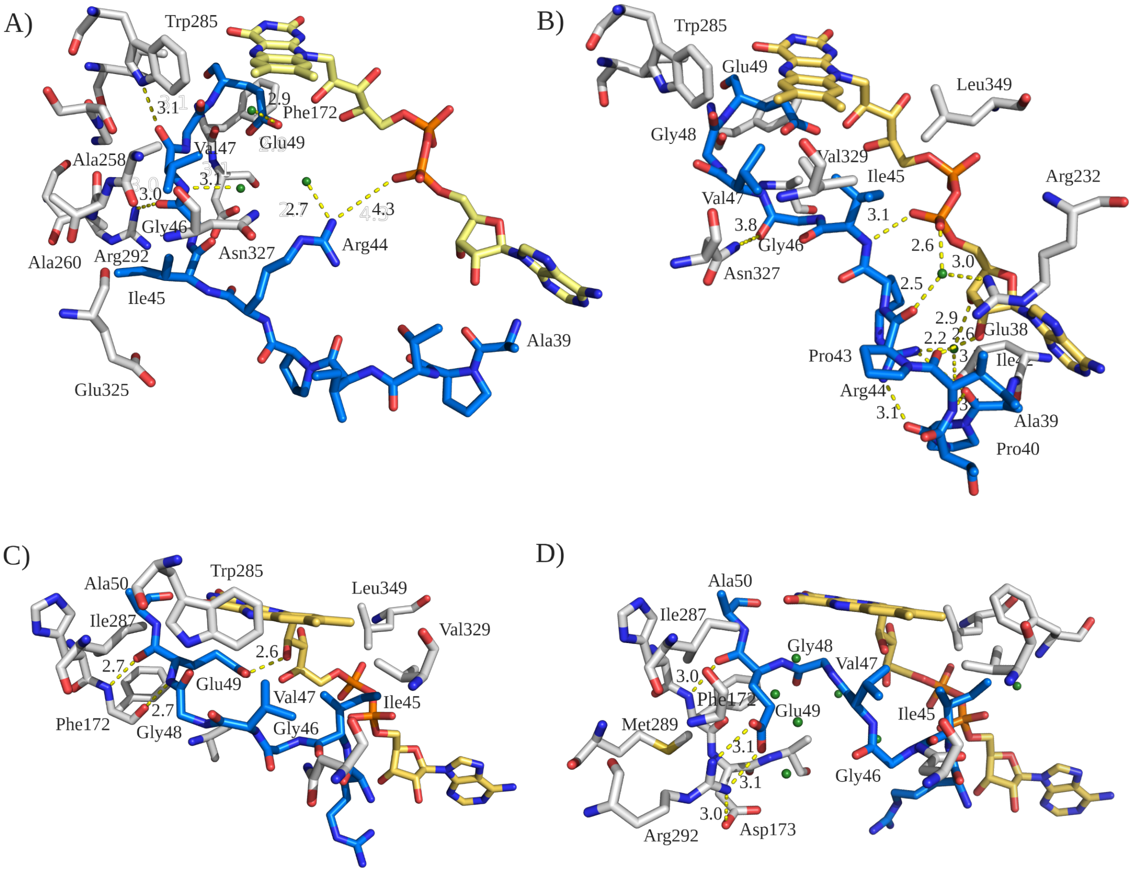

Figure S21. **Different conformations of the flavin binding loop.** The conformation of the residues 40-48 observed in Chain A of the Thal:FADH$^-$ complex (PDB code 7CU2) (loose conformation) (A). The conformation of the residues 40-48 observed in Chain B of the Thal:FADH$^-$ complex (PDB code 7CU2) (tight conformation) (B and C). The conformation of the residues 45-49 observed in Chain A of Thal:FAD:AMP (PDB code 7CU1) (open conformation) (D).

Molecular dynamics (MD) analysis to explain inactive complex formation.

Hydrogen atoms of amino acid residues were added to the Thal:FAD:AMP complex (PDB code 7CU1) by considering results from the propka (20). The atom types in the topology files were assigned based on the CHARMM27 parameter set (21). A structure of Thal was solvated in a cubic box of TIP3P with water extending at least 15 Å in each direction from the solute. Dimensions of the solvated system used in this study were 87 x 110 x 100 Å. MD parameters similar to previous work (36) were used in this analysis in order to understand the dynamics of bio-molecular systems. MD simulations were carried out using the NAMD program (37) with simulation protocols adapted from our previous work and NAMD tutorials (38,39). The simulations were started by minimizing hydrogen atom positions for 3,000 steps and followed by water minimization for 6,000 steps. The system water was heated to 300 K for 5 ps and then equilibrated for 15 ps. The whole system was minimized for 10,000 steps and heated to 300 K for 20 ps. Finally, the whole system was equilibrated for 180 ps and followed by a production stage for 4 ns.

REFERENCES

1. Schnepel, C., Minges, H., Frese, M., and Sewald, N. (2016) A High-Throughput Fluorescence Assay to Determine the Activity of Tryptophan Halogenases. *Angewandte Chemie International Edition* **55**, 14159-14163

2. Zeng, J., and Zhan, J. (2011) Characterization of a tryptophan 6-halogenase from Streptomyces toxytricini. *Biotechnology Letters* **33**, 1607-1613

3. Seibold, C., Schnerr, H., Rumpf, J., Kunzendorf, A., Hatscher, C., Wage, T., Ernyei, A. J., Dong, C., Naismith, J. H., and Van Pée, K.-H. (2006) A flavin-dependent tryptophan 6-halogenase and its use in modification of pyrrolnitrin biosynthesis. *Biocatalysis and Biotransformation* **24**, 401-408

4. Yeh, E., Cole, L. J., Barr, E. W., Bollinger, J. M., Ballou, D. P., and Walsh, C. T. (2006) Flavin Redox Chemistry Precedes Substrate Chlorination during the Reaction of the Flavin-Dependent Halogenase RebH. *Biochemistry* **45**, 7904-7912

5. Suadee, C., Nijvipakul, S., Svasti, J., Entsch, B., Ballou, D. P., and Chaiyen, P. (2007) Luciferase from Vibrio campbellii is more thermostable and binds reduced FMN better than its homologues. *The Journal of Biochemistry* **142**, 539-552

6. Pimviriyakul, P., Thotsaporn, K., Sucharitakul, J., and Chaiyen, P. (2017) Kinetic Mechanism of the Dechlorinating Flavin-dependent Monooxygenase HadA. *Journal of Biological Chemistry* **292**, 4818-4832

7. Sucharitakul, J., Chaiyen, P., Entsch, B., and Ballou, D. P. (2006) Kinetic Mechanisms of the Oxygenase from a Two-component Enzyme, p-Hydroxyphenylacetate 3-Hydroxylase from Acinetobacter baumannii. *Journal of Biological Chemistry* **281**, 17044-17053

8. Kantz, A., and Gassner, G. T. (2011) Nature of the Reaction Intermediates in the Flavin Adenine Dinucleotide-Dependent Epoxidation Mechanism of Styrene Monooxygenase. *Biochemistry* **50**, 523-532

9. Dong, C., Flecks, S., Unversucht, S., Haupt, C., van Pée, K.-H., and Naismith, J. H. (2005) Tryptophan 7-halogenase (PrnA) structure suggests a mechanism for regioselective chlorination. *Science* **309**, 2216-2219

10. Keller, S., Wage, T., Hohaus, K., Hölzer, M., Eichhorn, E., and van Pée, K.-H. (2000) Purification and Partial Characterization of Tryptophan 7-Halogenase (PrnA) from Pseudomonas fluorescens. *Angewandte Chemie International Edition* **39**, 2300-2302

11. Kirner, S., Hammer, P. E., Hill, D. S., Altmann, A., Fischer, I., Weislo, L. J., Lanahan, M., van Pée, K. H., and Ligon, J. M. (1998) Functions encoded by pyrrolnitrin biosynthetic genes from Pseudomonas fluorescens. *Journal of bacteriology* **180**, 1939-1943

12. Milbredt, D., Patallo, E. P., and van Pée, K.-H. (2014) A Tryptophan 6-Halogenase and an Amidotransferase Are Involved in Thienodolin Biosynthesis. *ChemBioChem* **15**, 1011-1020

13. Yeh, E., Blasiak, L. C., Koglin, A., Drennan, C. L., and Walsh, C. T. (2007) Chlorination by a Long-Lived Intermediate in the Mechanism of Flavin-Dependent Halogenases. *Biochemistry* **46**, 1284-1292

14. Zehner, S., Kotzsch, A., Bister, B., Süssmuth, R. D., Méndez, C., Salas, J. A., and van Pée, K.-H. (2005) A Regioselective Tryptophan 5-Halogenase Is Involved in Pyrroindomycin Biosynthesis in Streptomyces rugosporus LL-42D005. *Chemistry & Biology* **12**, 445-452

15. Senn, H. M. (2014) Insights into enzymatic halogenation from computational studies. *Front Chem* **2**, 98-98

16. Karabencheva-Christova, T. G., Torras, J., Mulholland, A. J., Lodola, A., and Christov, C. Z. (2017) Mechanistic Insights into the Reaction of Chlorination of Tryptophan Catalyzed by Tryptophan 7-Halogenase. *Scientific Reports* **7**, 17395

17. Ridder, L., Mulholland, A. J., Rietjens, I. M. C. M., and Vervoort, J. (2000) A Quantum Mechanical/Molecular Mechanical Study of the Hydroxylation of Phenol and Halogenated Derivatives by Phenol Hydroxylase. *Journal of the American Chemical Society* **122**, 8728-8738

18. Bitto, E., Huang, Y., Bingman, C. A., Singh, S., Thorson, J. S., and Phillips, G. N. (2008) The structure of flavin-dependent tryptophan 7-halogenase RebH. *Proteins: Structure, Function, and Bioinformatics* **70**, 289-293

19. Brunger, A. T., and Karplus, M. (1988) Polar Hydrogen Positions in Proteins: Empirical Energy Placement and Neutron Diffraction Comparison. *Proteins* **4**, 148 - 156

20. Dolinsky, T. J., Nielsen, J. E., McCammon, J. A., and Baker, N. A. (2004) PDB2PQR: an automated pipeline for the setup of Poisson–Boltzmann electrostatics calculations. *Nucleic Acids Res.* **32**, W665-W667

21. A. D. MacKerell, D. Bashford, M. Bellott, R. L. Dunbrack, J. D. Evanseck, M. J. Field, S. Fischer, J. Gao, H. Guo, S. Ha, D. Joseph-McCarthy, L. Kuchnir, K. Kuczera, F. T. K. Lau, C. Mattos, S. Michnick, T. Ngo, D. T. Nguyen, B. Prodhom, W. E. Reiher, B. Roux, M. Schlenkrich, J. C. Smith, R. Stote, J. Straub, M. Watanabe, J. Wirkiewicz-Kuczera, D. Yin, and Karplus, M. (1998) All-Atom Empirical Potential for Molecular Modeling and Dynamics Studies of Proteins. *J. Phys. Chem. B* **102**, 3586-3616

22. Reuter, N., Dejaegere, A., Maigret, B., and Karplus, M. (2000) Frontier Bonds in QM/MM Methods: A Comparison of Different Approaches. *J. Phys. Chem. A* **104**, 1720-1735

23. Wolfram Koch, M. C. H. (2001) *Chemist's Guide to Density Functional Theory*, Wiley-VCH, New York, NY, USA

24. Jensen, F. (2007) *Introduction to Computational Chemistry*, John Wiley & Sons, Chichester, UK

25. Becke, A. D. (1993) Density-functional thermochemistry. III. The role of exact exchange. *J. Chem. Phys.* **98**, 5648-5652

26. Hertwig, R. H., and Koch, W. (1997) On the parameterization of the local correlation functional. What is Becke-3-LYP? *Chem. Phys. Lett.* **268**, 345-351

27. Harvey, J. N. (2004) Spin-forbidden CO ligand recombination in myoglobin. *Faraday Discuss.* **127**, 165-177

28. Frisch, M. J., Trucks, G. W., Schlegel, H. B., Scuseria, G. E., Robb, M. A., Cheeseman, J. R., Scalmani, G., Barone, V., Mennucci, B., Petersson, G. A., Nakatsuji, H., Caricato, M., Li, X., Hratchian, H. P., Izmaylov, A. F., Bloino, J., Zheng, G., Sonnenberg, J. L., Hada, M., Ehara, M., Toyota, K., Fukuda, R., Hasegawa, J., Ishida, M., Nakajima, T., Honda, Y., Kitao, O., Nakai, H., Vreven, T., Montgomery, J. A., Peralta, J. E., Ogliaro, F., Bearpark, M., Heyd, J. J., Brothers, E., Kudin, K. N., Staroverov, V. N., Kobayashi, R., Normand, J., Raghavachari, K., Rendell, A., Burant, J. C., Iyengar, S. S., Tomasi, J., Cossi, M., Rega, N., Millam, J. M., Klene, M., Knox, J. E., Cross, J. B., Bakken, V., Adamo, C., Jaramillo, J., Gomperts, R., Stratmann, R. E., Yazyev, O., Austin, A. J., Cammi, R., Pomelli, C., Ochterski, J. W., Martin, R. L., Morokuma, K., Zakrzewski, V. G., Voth, G. A., Salvador, P., Dannenberg, J. J., Dapprich, S., Daniels, A. D., Farkas, Foresman, J. B., Ortiz, J. V., Cioslowski, J., and Fox, D. J. (2009) Gaussian 09, Revision B.01. Wallingford CT

29. Ponder, J. W. (2003) *TINKER:Software Tools for Molecular Design*, v4.0 ed., MO, Saint Louis

30. Lawan, N., Chasing, P., Santatiwongchai, J., and Muangpil, S. (2019) QM/MM molecular modelling on mutation effect of chorismate synthase enzyme catalysis. *Journal of Molecular Graphics and Modelling* **87**, 250-256

31. Lawan, N., Ranaghan, K. E., Manby, F. R., and Mulholland, A. J. (2014) Comparison of DFT and ab initio QM/MM methods for modelling reaction in chorismate synthase. *Chemical Physics Letters* **608**, 380-385

32. Ranaghan, K. E., Morris, W. G., Masgrau, L., Senthilkumar, K., Johannissen, L. O., Scrutton, N. S., Harvey, J. N., Manby, F. R., and Mulholland, A. J. (2017) Ab Initio QM/MM Modeling of the Rate-Limiting Proton Transfer Step in the Deamination of Tryptamine by Aromatic Amine Dehydrogenase. *The Journal of Physical Chemistry B* **121**, 9785-9798

33. van der Kamp, M. W., Żurek, J., Manby, F. R., Harvey, J. N., and Mulholland, A. J. (2010) Testing High-Level QM/MM Methods for Modeling Enzyme Reactions: Acetyl-CoA Deprotonation in Citrate Synthase. *The Journal of Physical Chemistry B* **114**, 11303-11314

34. Werner, H.-J., Knowles Peter, J., Knizia, G., Manby Frederick, R., and Schütz, M. (2011) Molpro: a general-purpose quantum chemistry program package. *Wiley Interdiscip. Rev. Comput. Mol. Sci.* **2**, 242-253

35. Olsson, M. H. M., Søndergaard, C. R., Rostkowski, M., and Jensen, J. H. (2011) PROPKA3: Consistent Treatment of Internal and Surface Residues in Empirical pKa Predictions. *Journal of Chemical Theory and Computation* **7**, 525-537

36. Karplus, M., and Petsko, G. A. (1990) Molecular dynamics simulations in biology. *Nature* **347**, 631

37. J. C. Phillips, R. Braun, W. Wang, J. Gumbart, E. Tajkhorshid, E. Villa, C. Chipot, R. D. Skeel, L.Kalé, and Schulten, K. (2005) Scalable molecular dynamics with NAMD. *J. Comp. Chem.* **26**, 1781 - 1802

38. J. Phillips, T. Isgro, M. Sotomayor, E. Villa, H. Yu, D. Tanner, and Y. Liu. (2012) NAMD tutorial.

39. T. Isgro, J. Phillips, M. Sotomayor, E. Villa, H. Yu, D. Tanner, Y. Liu, Wu, Z., and D. Hardy. (2017) NAMD tutorial.
